# Is treatment-resistant schizophrenia categorically distinct from treatment-responsive schizophrenia? a systematic review

**DOI:** 10.1186/s12888-016-1177-y

**Published:** 2017-01-13

**Authors:** Amy L. Gillespie, Ruta Samanaite, Jonathan Mill, Alice Egerton, James H. MacCabe

**Affiliations:** 1 Psychosis Studies Department, Institute of Psychiatry, Psychology and Neuroscience, King’s College London, 16 De Crespigny Park, Denmark Hill, London, SE5 8AF UK; 2 Social, Genetic and Developmental Psychiatry Centre, Institute of Psychiatry, Psychology and Neuroscience, King’s College London, London, UK; 3 University of Exeter Medical School, Exeter University, St Luke’s Campus, Exeter, UK

**Keywords:** Schizophrenia, Treatment resistance, Treatment refractory, Treatment response, Clozapine, Classification

## Abstract

**Background:**

Schizophrenia is a highly heterogeneous disorder, and around a third of patients are treatment-resistant. The only evidence-based treatment for these patients is clozapine, an atypical antipsychotic with relatively weak dopamine antagonism. It is plausible that varying degrees of response to antipsychotics reflect categorically distinct illness subtypes, which would have significant implications for research and clinical practice. If these subtypes could be distinguished at illness onset, this could represent a first step towards personalised medicine in psychiatry. This systematic review investigates whether current evidence supports conceptualising treatment-resistant and treatment-responsive schizophrenoa as categorically distinct subtypes.

**Method:**

A systematic literature search was conducted, using PubMed, EMBASE, PsycInfo, CINAHL and OpenGrey databases, to identify all studies which compared treatment-resistant schizophrenia (defined as either a lack of response to two antipsychotic trials or clozapine prescription) to treatment-responsive schizophrenia (defined as known response to non-clozapine antipsychotics).

**Results:**

Nineteen studies of moderate quality met inclusion criteria. The most robust findings indicate that treatment-resistant patients show glutamatergic abnormalities, a lack of dopaminergic abnormalities, and significant decreases in grey matter compared to treatment-responsive patients. Treatment-resistant patients were also reported to have higher familial loading; however, no individual gene-association study reported their findings surviving correction for multiple comparisons.

**Conclusions:**

Tentative evidence supports conceptualising treatment-resistant schizophrenia as a categorically different illness subtype to treatment-responsive schizophrenia. However, research is limited and confirmation will require replication and rigorously controlled studies with large sample sizes and prospective study designs.

**Electronic supplementary material:**

The online version of this article (doi:10.1186/s12888-016-1177-y) contains supplementary material, which is available to authorized users.

## Background

Schizophrenia is highly heterogeneous and better characterisation of this heterogeneity is needed to progress research into aetiology, mechanisms and treatment. While the majority of patients with schizophrenia respond to typical or atypical non-clozapine antipsychotics, roughly a third of patients do not respond well and are considered treatment-resistant [1]. Differential treatment response represents a discontinuity which could help divide schizophrenia into biologically distinct subtypes, as Farooq et al. suggest [2]. If these subtypes could be distinguished using genetic or other markers, this would be a significant step towards the introduction of personalised medicine in schizophrenia treatment. This paper will review the literature as to whether treatment-resistant and treatment-responsive schizophrenia reflect categorically distinct subtypes.

### Treatment-resistant schizophrenia

Evidence suggests that treatment-resistance is a stable trait, as an early lack of response to treatment has been consistently shown to predict poor treatment outcome and diagnosis of treatment-resistance [3–5]. First described in the 1988 Kane et al. criteria [6], a consistent minimum requirement for a diagnosis of treatment-resistance is two periods of treatment with different antipsychotics at adequate dose (variously defined), each for at least 4 weeks, without at least a 20% reduction in symptoms. This is reflected in guidelines for clozapine prescription [7, 8]: a 2014 review of clozapine prescription trends concludes that clozapine has consistently remained the gold standard for treatment-resistant schizophrenia, with all evidence-based guidelines recommending prescription “after failure of two adequate trials of two different antipsychotic agents” [9]. Recent variations have progressed from exclusively considering persistent positive symptoms to also incorporating persistent negative and cognitive symptoms [10, 11]; however positive symptoms remain a central focus as the main target of antipsychotics and the primary outcome in the early clozapine trials which defined treatment-resistance (see 2016 review highlighting role of positive symptoms [12]).

The dopamine hypothesis [13] is arguably the most well-known and well-supported neurochemical model of schizophrenia, but has been unable to explain the occurrence of treatment-resistant schizophrenia. While clinical response to antipsychotics is strongly correlated with dopamine receptor D2 occupancy for most patients, treatment-resistant patients show no clinical response even when their D2 receptor occupancy is above the therapeutic threshold [14]. Furthermore, clozapine is found to be highly effective in treatment-resistant patients [6], despite relatively low levels of D2 receptor occupancy [15]. It has been suggested that systematic differences may underlie this differential response of patients to antipsychotics [16]. In particular, that the dopamine hypothesis may not apply to treatment-resistant schizophrenia [17], where symptoms are instead driven by non-dopaminergic abnormalities, perhaps involving the glutamate system [18–20].

### Burden of treatment-resistance

Approximately a third of patients are treatment-resistant [1, 8], though some estimates are as high as 40–60% [21, 22]. Among mental illness, treatment-resistant schizophrenia is associated with some of the highest levels of impaired functioning [23], rates of hospitalisation [24], and costs to society, with one review estimating that treatment-resistance leads to an additional $34 billion in direct healthcare costs in the United States alone [25].

As Farooq et al. comment [2], classifying schizophrenia by treatment-response helps ensure that treatment-resistance remains a priority; they propose establishing clinical criteria that distinguishes these groups and then evaluating the endophenotypes and biomarkers present in these samples. Previous reviews have looked at predictors of treatment-response in schizophrenia [16], but we are unaware of any reviews that have focused exclusively on studies comparing patients with treatment-resistance (defined as above by two failed antipsychotic trials) against known treatment-responders. Therefore, we conducted a systematic review of all studies which compare treatment-resistant versus treatment-responsive patients with schizophrenia to investigate whether current evidence supports a conceptualisation of these as categorically distinct subtypes.

## Method

### Inclusion/exclusion criteria

Our inclusion criteria were: original data comparing patients with treatment-resistant and treatment-responsive schizophrenia; all patients have a primary diagnosis of schizophrenia or schizoaffective disorder; the definition of treatment-resistance incorporates (as a minimum) two failed antipsychotic trials; the treatment-responsive group comprises of patients with a known response to non-clozapine antipsychotics; publication in a peer-reviewed journal.

Studies were excluded if: treatment-resistance was classified based on only one antipsychotic trial; treatment-responsiveness was defined solely as not meeting treatment-resistant criteria; the group labelled “treatment-responsive” included patients on clozapine; if studies of clinical variables were cross-sectional (as treatment-resistance is defined by current clinical state).

### Search strategy

The databases searched were: Ovid (on 25^th^ January 2015, including Medline, Embase, PsycInfo and PsycArticle), PubMed (on 2^nd^ February 2015) and CINAHL (on 2^nd^ February 2015). The grey literature database OpenGrey was also searched on 2^nd^ February 2015. Search terms included synonyms for schizophrenia and treatment-resistance (Additional file 1). Relevant review articles and conference proceedings published between 2005 and 2015 were identified and reference and abstract listings were screened. All eligible studies were forward and backward screened. At the stage of full-text screening, the two journals with the highest number of potentially eligible publications were Biological Psychiatry and Schizophrenia Research; therefore, the tables of contents for all online volumes of these journals were hand-searched. Authors of all eligible studies were contacted requesting any other eligible findings, published or unpublished.

All identified studies underwent title and abstract screening, then all potentially eligible studies underwent full-text screening. When full-text articles were not available or eligibility was unclear, authors were contacted where possible. If confirmation of eligibility was not possible, studies were excluded. AG did the initial screening, then RS independently re-screened a randomly selected a) 10% of titles and abstracts identified by the database search and b) 20% of full-text articles identified by title and abstract screening. Cohen’s kappa was calculated to provide a conservative measure of inter-rater reliability; this produced kappas of 0.8 and 1 respectively, indicating substantial agreement. If the second reviewer identified novel potentially eligible studies, these were re-screened collaboratively.

A standard data extraction form was used (Additional file 1). If the treatment-resistant group were sub-divided by clozapine response, the data from both the clozapine-responsive and clozapine-nonresponsive (known as ultra-treatment resistant) group were reported.

### Quality assessment

To assess study quality and risk of bias, the Newcastle-Ottawa Scale for case-control studies was adapted (Additional file 1), as has been done previously [26]. The scale was adapted in advance to suit the scope of the research question, to include items on statistical analysis and sample size, and to allow for more differentiation between studies. Studies were scored out of 5 for selection of participants, out of 2 for comparability and out of 4 for predictor ascertainment and analysis. A total score of 9 or above was deemed high quality, a score of 6–8 was deemed moderate quality, and below 5 was deemed low quality. Low quality studies were then excluded from the review.

This systematic review has been conducted and reported according to PRISMA principles and guidelines, to aid evaluation and utilisation (see Additional file 2: PRISMA checklist).

## Results

**Fig. 1:**
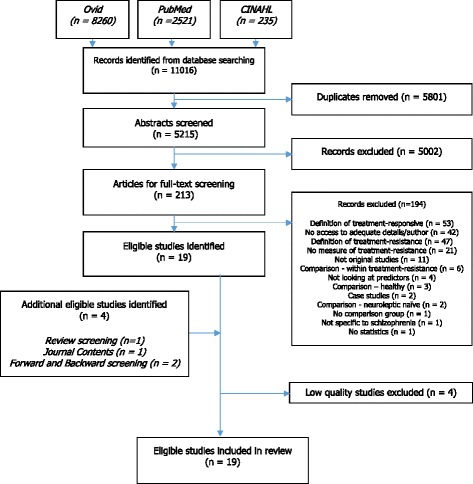
Flowchart of identified, included and excluded studies

From 5215 unique studies identified by the database search, 19 papers met inclusion criteria. 4 more eligible studies were identified through further screening methods outlined above. 4 were excluded due to low quality scores. (See Fig. 1 for flowchart and Additional file 1 for excluded studies table).

Overall, 19 studies are included in this review (Additional file 1). Two studies had an overlap in participants but report on different variables.^1^ The average quality assessment score (Additional file 1) was 6.9 (range 6–8), moderate: 3.2 for selection, 0.3 for comparability and 3.4 for predictors. There were no studies with a high quality score; as low quality studies were excluded, this means all studies were of moderate quality. Definitions for treatment-resistance and response varied considerably, as shown in Tables 1 and 2.

**Table 1:** Definitions of treatment-resistance

Study	Chlorpromazine dose and trial length	Number of trials necessary	Minimum length of illness	Minimum severity score	Specify symptoms	Clozapine prescription	Responsive to clozapine	Inability to live independently	Prospective confirmation
28	“Therapeutic doses” 6 weeks	2				x			
29	“Adequate” 8 weeks	2		CGI ≥ 4	>4 on 1+ specified PANSS item				
17	400–600 4–6 weeks	2	5 years	BPRS > 45	>4 on 2+ specified BPRS items			x	
18	400–600 4–6 weeks	2	5 years	BPRS > 45	>4 on 2+ specified BPRS items			x	
27	NICE algorithm			CGI < 4		x			
30	400 mg 4 weeks	2		CGI > 4	One of specified BPRS items				
33	400 mg 4 weeks	2		CGI ≥ 4	One of specified BPRS items	x			
34	“sufficient duration”	“several”		May score 5–7				x	
32	“optimal clinical requirements”	3	Continuous for 2 years					x	
35	400 mg 4 weeks	2		CGI ≥ 4	One of specified BPRS items	x			
36	“sufficient duration”	“several”						x	
31	“sufficient duration”	“several”						x	
37	1000 mg 6 weeks	3		BPRS > 45 CGI > 4	>4 on 2+ specified BPRS items	x	x		
38	1000 mg 8 weeks	3							
39	750 mg 6 weeks	3	Continuous for 2 years	GAS < 40				x	
40	4–6 weeks	2							<30% decrease in PANSS
41	Kane criteria					inclusive	inclusive		
42	Therapeutic doses 6 weeks	2							
43	750 mg 6 weeks	3	2 years	BPRS > 45 CGI > 4

**Table 2:** Definitions of treatment-responsiveness

Study	Consistent good response to antipsychotics	Remission allowing discharge	Remission sustained over long period	Minimum severity before treatment	Minimum improvement	Maximum severity after treatment	Relapse when medication discontinued	Prospective confirmation	Other
28					>50% decrease in BPRS/PANSS				
29									x
17			6 months		x	≤3 PANSS item scores; ≤3 BPRS item scores; ≤2 SAPS/SANS item scores			
18			6 months		x	≤3 PANSS item scores; ≤3 BPRS item scores; ≤2 SAPS/SANS item scores			
27						At most mildly ill (CGI)			
30			x	CGI > 4					
33			x	CGI > 4		No psychotic symptoms			
34		x		May score 1–4					
32	x			at least 1 admission	“full or partial remission”				
35			x	CGI > 4		No psychotic symptoms			
36									x
31		x							
37					20% decrease in BPRS total	CGI rating ≤3 or BPRS score ≤ 35			
38		x			CGI rating of “very/much improved”			12 weeks haloperidol < 30 mg	
39	x			at least 1 admission	“full or partial remission”	CGI rating ≤3 or BPRS score ≤ 30	x		x
40					>30% decrease in PANSS			x	
41						“at most mild”			
42									x
43	x			at least 1 admission		BPRS score ≤ 30 CGI rating <3	x		

### Neuroimaging

There were five neuroimaging studies covering magnetic resonance imaging (MRI), electroencephalography (EEG), positron-emission tomography (PET), and magnetic resonance spectroscopy (MRS). All report controlling for basic demographics like age and sex but only three [17, 18, 27] report controlling for other confounders such as ethnicity, weight and smoking.

Two investigated brain volume, measured by MRI. In a study of 37 New Zealand patients (19 resistant, 18 responsive), Anderson et al. reported significantly lower grey matter volumes, especially in cortical areas, for treatment-resistant patients, but no differences in whole brain or white matter volume [28]. Ultra-treatment resistant patients also showed significantly lower grey matter volume than treatment-responsive patients, with numerically larger deficits than treatment-resistant patients (though this difference was not significant). Compared to healthy volunteers, treatment-responsive patients did not significantly differ in grey matter volume, while treatment-resistant patients showed significantly lower volumes. All the treatment-resistant patients were prescribed clozapine, while the majority of treatment-responsive patients were prescribed olanzapine or risperidone. In Molina et al., 49 Spanish patients (30 resistant, 19 responsive) were studied [29]; no patients had taken clozapine prior to baseline assessments, and all patients were trialled on haloperidol to confirm resistance or responsiveness. They report that discriminant analysis demonstrated hypertrophy in occipital white matter to be the best predictor of treatment-resistance, and that significant decreases in frontal and occipital grey matter of treatment-resistant patients compared to healthy volunteers were not evident in treatment-responsive patients.

In the same study, Molina et al. also investigated P300 parameters - the late component of the event-related potential - with EEG but found no differences between responsive and resistant patients [29].

One study compared striatal dopamine synthesis capacity with PET measurement of [18 F]-DOPA uptake, in a matched-design study of 24 British patients (12 resistant, 12 responsive) [17]. No participants were prescribed clozapine at the time of scanning (though two treatment-resistant patients had been previously prescribed clozapine but experienced adverse reactions); the majority of patients in both groups were prescribed olanzapine or risperidone depot. They reported (with multiple testing correction) significantly lower dopamine synthesis capacity in treatment-resistant patients compared to treatment-responsive patients, in the whole striatum; and in the associative and limbic, but not sensorimotor, striatal subdivisions. Compared to healthy volunteers, treatment-resistant patients were not significantly different, unlike treatment-responsive patients who showed significantly higher dopamine synthesis capacity than healthy volunteers.

Two studies measured regional brain metabolite concentrations using MRS. Demjaha et al. [18] carried out a study of 14 patients (6 resistant, 8 responsive) and found that treatment-responders had significantly lower levels of N-acetyl aspartate (NAA) in the anterior cingulate cortex (ACC) than both treatment-resistant patients and healthy volunteers. Also, while ACC glutamate levels in treatment-resistant patients compared to treatment-responsive patients were not significantly different, treatment-resistant patients had significantly elevated ACC glutamate levels compared to healthy volunteers, while treatment-responsive patients did not. Medication status or history was not reported. Goldstein et al. [27] performed a MRS study in 31 New Zealand patients (16 resistant, 15 responsive), acquiring data in the putamen, dorsolateral prefrontal cortex (DLPFC) and ACC. In contrast to Demjaha et al., they found no group differences in glutamate, NAA or choline. However, they did find patients with treatment-resistant schizophrenia had significantly higher total glutamate + glutamine (Glx) in the putamen (with family-wise error correction) than both treatment-responsive and ultra-treatment resistant patients. There was no significant difference between healthy volunteers and treatment-resistant patients, but healthy volunteers appeared to have very similar levels to the two other patient groups. Also, ultra-treatment resistant patients had significantly lower Glx in the DLPFC than treatment-responsive patients, with treatment-resistant patients and healthy volunteers showing levels in between. All their treatment-resistant patients were prescribed and responsive to clozapine, while all treatment-responsive patients were prescribed non-clozapine atypical antipsychotics (with the majority prescribed olanzapine and risperidone).

### Gene-association

There were nine gene-association studies, with no eligible genome-wide association study (GWAS) papers. All of them used white participants.

Two investigated polymorphisms in the brain-derived neurotrophic factor (BDNF) gene. In a study of 94 Finnish patients (51 resistant, 43 responsive), no group differences were found for the *G169A polymorphism (Val66Met)* or the *C270T polymorphism* [30]. In a study of 88 French patients (20 resistant, 68 responsive), treatment-resistant patients displayed significantly fewer long alleles of the BDNF *dinucleotide repeat polymorphism* than treatment-responsive patients [31].

Two studied the serotonin 2A receptor (5-HT2A) gene, specifically the *T102C polymorphism*. In a study of 102 Canadian patients (63 resistant, 39 responsive), a significantly higher frequency of the C/C genotype in treatment-resistant patients was reported [32] and a study of 94 Finnish patients (51 resistant, 43 responsive) replicated this association, but only in females [33].

The latter also reported a significantly higher frequency of the C/A genotype for the tryptophan hydroxylase enzyme (TPH1) gene in treatment-resistant patients, but no group differences for the guanine-nucleotide-binding protein (GNB3) gene [33].

In a study of 193 French patients (45 resistant, 148 responsive) considering the *5’UTR (ccG repeat) polymorphism* of the reelin gene, a significantly higher frequency of ccG10 alleles in treatment-resistant patients was found [34].

Another study investigated the regulator of g-protein signalling (RGS4) gene in a study of 93 Finnish patients (50 resistant, 43 responsive), but found no group differences [35].

The dopamine receptor 3 (DRD3) gene, specifically the *Bal I polymorphism*, was examined in a study of 89 French patients (19 resistant, 70 responsive) which reported significantly less homozygosity for treatment-resistant patients [36].

A study of 38 Finnish patients (19 resistant, 19 responsive) investigated the human leukocyte antigen (HLA) genotypes and found significantly higher *HLA-A1 allele* frequency for treatment-resistant patients [37]. In contrast, a study of 88 Israeli patients (50 resistant, 38 responsive) conducted HLA typing and found no group difference in frequencies of different classes of antigens [38]. All treatment-resistant patients were prescribed clozapine, while all treatment-responsive patients were prescribed haloperidol.

Unfortunately, none of these findings are reported to survive multiple-testing correction: four do not report correction [31, 33, 34, 36] and two report their findings becoming non-significant after correction [32, 37].

### Genetic loading

There was one study of familial genetic loading and prevalence in relatives, which studied the prevalence of schizophrenia spectrum disorders, cluster A personality disorders and long-term psychiatric care in relatives, and calculated family loading scores and morbidity risks [39]. In a study of 71 Canadian patients (35 resistant, 36 responsive), first and second degree relatives of treatment-resistant patients had a significantly higher morbidity risk of schizophrenia spectrum disorders compared to relatives of treatment-responsive patients, and a significantly higher familial-loading score was found for treatment-resistant patients. While treatment-resistant patients demonstrated significantly greater risk than healthy volunteers, treatment-responsive patients did not differ significantly. No group differences were found for risk of cluster A personality disorders or long-term psychiatric care in relatives.

### Clinical variables

Two studies investigated whether clinical variables were associated with subsequent treatment response or resistance.

There was one prospective study [40] of Positive and Negative Syndrome Scale (PANSS) scores^2^ in which patients with no previous regular antipsychotic use and an illness onset within the last five years were randomised to receive either first or second generation non-clozapine antipsychotics in an open trial. Those that did not respond after one trial were then switched to another antipsychotic, and those that failed two trials were deemed treatment-resistant (4 patients in total). In the study of 17 Brazilian patients, treatment-resistance at twelve weeks was predicted by lower baseline total PANSS score. This study also measured improvement in the first 2 weeks of antipsychotic treatment and did not find this to be predictive of treatment-resistance.

Meltzer et al. studied age of onset [41]. In a study of 322 American patients (196 resistant, 126 responsive), treatment-resistant patients had an earlier age of onset.

### Neurocognitive function

There were two papers looking at neurocognitive function.

Joober et al. examined 75 Canadian patients (39 resistant, 36 responsive) [42]. Treatment-resistant patients had greater deficits in verbal ability and language, verbal memory and learning, and visual memory, but there were no significant differences for visual-spatial ability, abstraction and concept formation, visual motor processing and sustained attention. Treatment-resistant patients were prescribed a combination of typical and atypical antipsychotics (including clozapine), while all but four treatment-responsive patients were prescribed typical antipsychotics.

De Bartolomeis et al. investigated verbal memory, working memory, motor speed, verbal fluency, processing speed and executive functions, in a study of 41 Italian patients (19 resistant, 22 responsive) [43]. The only significant difference reported was lower verbal memory scores in treatment-resistant patients. Medication status or history separated by group is not reported but prescription of first and second-generation antipsychotics was not significantly different between treatment-resistant and responsive patients.

### Demographic characteristics

There was one study measuring demographic characteristics. Meltzer et al.– described above - studied gender and ethnicity [41]. The study demonstrated a significantly higher frequency of white patients in the treatment-resistant group compared to treatment-responsive, but no gender difference.

## Discussion

### Strengths and weaknesses

This is the first systematic review directly addressing whether treatment-resistant schizophrenia is categorically different from treatment-responsive schizophrenia, a question with substantial implications for research and clinical practice. It is somewhat unusual for a systematic review to address a theoretical question like this, but the benefits of comprehensiveness, reduced bias, and replicability remain desirable. To ensure that findings were not biased towards research fields that unduly influenced the conclusions, a broader search strategy than is conventional was necessary. This provides a comprehensive overview allowing the emergence of a pattern across research fields. It also highlights the areas lacking in quantity or quality of research. Another strength of this review is the focused definitions of treatment-resistance and treatment-responsiveness; they relate to clinical practice for diagnosing and treating treatment-resistance and provide a comparison between two groups of patients with distinctly different responses to antipsychotics.

However, this study does have limitations. Firstly, while every effort was made to seek out all available research there is a stronger chance of publication bias for non-RCT studies, as there are less protective measures (such as prospective registration of trials) in place; however, we did identify several studies reporting null findings (e.g., 4 out of 9 gene-association studies reported null results). Secondly, despite not excluding non-English language papers, papers were largely Western and participants were largely white. Thirdly, significant heterogeneity remained in definitions of treatment-resistance and treatment-responsiveness and in the populations and methods used to recruit participants, making comparison different. Fourthly, in comparing based on treatment outcome, antipsychotic response and resistance are not readily separable from group differences in illness symptomatology or severity. Also, the binary comparison of treatment-resistant patients to confirmed treatment-responders arguably ignores some of the complexity of the disorder. Not only can treatment-response be considered along a spectrum [44], but schizophrenia is heterogenous in more than just treatment-outcome and likely encompasses a spectrum of disorders [45]. However, focusing on this comparison of patient groups with distinctly different responses to antipsychotics is a strong starting point for addressing the heterogeneity, one likely to provide larger effect sizes and one which provides information with significant clinical relevance. Finally, the strict definitions of treatment-resistant and treatment-responsive patients mean that potentially relevant studies investigating markers of treatment-resistance using different definitions have been excluded. The rest of the discussion incorporates particularly pertinent studies from this broader literature.

### Neurotransmission

#### Dopamine system

Two studies identified in this review indicate that treatment-resistant schizophrenia may be differentiated from treatment-responsive schizophrenia by measures of dopaminergic function: specifically, lower levels of striatal dopamine synthesis capacity, equivalent to levels in healthy volunteers [17], and less homozygosity of the DRD3 allele [36]. As the dopamine synthesis study was cross-sectional, the group difference may reflect differences in medication history or other extraneous group differences. However, the finding of lower dopamine synthesis in treatment-resistant patients is consistent with evidence that elevated levels of baseline synaptic dopamine and plasma levels of homovanilic acid (HVA) – a metabolite of dopamine - predict good treatment response [46, 47] (these studies were not included in our review as they looked at response to a single antipsychotic trial). This finding also aligns with two relevant reviews: one review of predictors of antipsychotic response (primarily citing studies of single antipsychotic trials) that concluded HVA was the most predictive peripheral marker [16], and one systematic review of neuroimaging findings in resistant and responsive patients with schizophrenia (without our strict definitions of these groups) which highlighted reduced striatal dopamine synthesis as one of five features of treatment-resistant schizophrenia [48].

If these findings reflect that treatment-resistant patients lack the striatal dopaminergic elevations typically detected in schizophrenia, this could explain why treatment-resistant patients show little response to D2 dopamine receptor blockade with conventional antipsychotic treatment [14]. This would implicate categorical differences between the two patient groups – one displaying normal dopamine function and one displaying abnormalities – rather than a difference of severity, and this is also the conclusion of another systematic review [48].

#### Serotonin system

The studies identified report that treatment-resistance may be differentiated from treatment-responsive schizophrenia by predominance of the C allele of the T102C polymorphism of the 5HT2A gene (encoding the main excitatory serotonin receptors) [32, 33] and predominance of the C/A genotype for the A779C of the TPH1 gene (encoding the enzyme that catalyses the conversion of tryptophan to 5-HTP, serotonin’s precursor) [33]. Genetic abnormalities driving a categorically different pattern of serotonin function in treatment-resistant patients would be consistent with the fact that clozapine is a potent 5-HT2A receptor antagonist [49]. However, while the 5HT2A association was the only replicated gene-association finding in our review, neither study survived multiple-testing correction and reviews on T102C and treatment-response find largely null results [16]. Also, the A779C polymorphism has been associated with nicotine dependence creating a possible confounder [50].

Nonetheless, other studies (not included in our review as they looked at response to only one antipsychotic trial) have found that lower 5-HT plasma levels, as well as a subsequent increase during treatment, predict poorer response to atypical antipsychotics, with responders showing equivalent levels to healthy volunteers [51]. Similarly, those who respond to D-fenfluramine (which enhances serotonin transmission) with greater serotonin release have been shown to respond poorly to subsequent antipsychotic treatment [52].

#### Glutamate system

The identified studies indicate that treatment-resistance may be differentiated by abnormalities in brain glutamate concentrations not seen in treatment-responsive patients; as with the dopamine findings, this again suggests a categorical difference rather than one of severity. A study by our group found higher levels of glutamate in the ACC in treatment-resistant but not treatment-responsive patients compared to healthy volunteers [18]. We have recently (published after the searches in this review were conducted) reported elevated ACC glutamate in resistant compared to responsive patients in a separate sample [20], and the results are also consistent with a study in first-episode patients showing that those patients who respond poorly to treatment have elevated ACC glutamate levels compared to those who respond well [19]. Together these studies suggest that elevated ACC glutamate may associate with poor antipsychotic response. However, Goldstein et al. [27], found no group differences in glutamate in the ACC, although glutamate plus glutamine (Glx) was elevated in the putamen in the treatment-resistant compared to responsive group, with the responsive group appearing equivalent to healthy volunteers. When subdividing within treatment-resistant patients this elevated Glx was specific to those who responded to clozapine, and not demonstrated in ultra-treatment resistant patients, suggesting a role in clozapine’s efficacy.

Other reviews of neuroimaging studies of treatment-resistance have differed in their interpretation of this literature, with a shift towards confidence that glutamatergic differences exist. One 2015 review [53] reported the Demjaha et al. findings but concluded there were no consistent findings in the field, whereas the 2016 Mouchlianitis et al. review [48] reported both papers included in the present review and highlighted both elevated glutamate in the ACC and elevated Glx in the putamen of clozapine responders as features of treatment-resistance.

One possible mechanism underlying the efficacy of clozapine in treatment-resistant illness may relate to its’ ability to attenuate glutamate release, as demonstrated in several rat studies [54, 55]. The study of Demjaha et al., also reported lower concentrations of NAA, generally viewed as a marker of neuronal integrity, in the ACC of treatment responsive compared to treatment-resistant patients [18]; however, this finding has not been replicated in other studies [20, 27].

#### Brain structure

Studies identified by this review find that treatment-resistant patients have reduced grey matter volumes in comparison to treatment-responsive patients [28, 29]. There is evidence for structural brain differences across patients with schizophrenia [56, 57], but both of the studies identified in the present review found that it was only treatment-resistant patients that showed a significant reduction in grey matter compared to healthy volunteers; treatment-responsive patients showed no significant differences in grey matter compared to healthy volunteers. This suggests that abnormal reductions in grey matter may be specifically relevant to treatment-resistant patients, and not an example of simply more severe abnormalities. However, when subdividing within treatment-resistant patients it appears that more severe reductions may be found in ultra-treatment resistant patients [28] (though this was not statistically significant).

Despite evidence that clozapine induces distinctive structural changes in grey matter [58], one study included treatment-resistant patients on clozapine while the other did baseline imaging before their first dose of clozapine, suggesting that this pattern should not immediately be dismissed as secondary to clozapine treatment. This pattern corroborates previous studies (not included as they only look at response to a single antipsychotic trial) which have found that smaller grey matter volume is associated with poorer response to haloperidol [59] and a prospective study in first-episode patients finding reduced occipital grey matter predicted lack of response [60].

These findings are consistent with the two other reviews of treatment-resistant vs treatment-responsive patients [48, 53] which identified studies demonstrating reduced grey matter; however again, the 2015 review is more cautious stating no consistent findings based on the two they discuss, while the 2016 review identifies seven studies and thus highlights reduced grey matter (particularly in frontal regions) as one of the features of treatment-resistance. The latter also suggest that these structural differences could be caused by high concentrations of glutamate, providing a plausible link between these findings within the neuroimaging literature.

In contrast to our discussion above, Mouchlianitis et al. conclude that grey matter abnormalities appears to be a difference of degree along a continuum [48]; however, this may be explained by their use of a much broader definition of treatment-resistance and responsiveness. Perhaps consistent treatment-responders show a distinct lack of these abnormalities, but non-resistant patients (who may later develop resistance, or show minimal response and fall between the two groups of patients) show a reduction in grey matter volume that falls along a continuum with consistent treatment-resistant patients and ultra-treatment resistant patients at the extremes.

Regarding white matter, Molina et al. [29] report that increased occipital white matter at baseline was predictive of subsequent treatment-resistance, which contrasts with the null results reported in Anderson et al. [28] in patients already taking clozapine. Mouchlianitis et all identify the same two studies, noting that greater white matter is similarly reported in the latter study but the difference between patient groups doesn’t reach significance [48]. One possibility is that clozapine may work to normalise white matter abnormalities, as studies have demonstrated reductions in white matter in patients taking clozapine [61].

#### Genes involved in neural development/growth

##### BDNF

Studies in this review indicate that treatment-resistant schizophrenia may be differentiated from treatment-responsive schizophrenia by shorter alleles of the dinucleotide repeat polymorphism [31], but not the G196A (Val66Met) or C270T polymorphism [30]. However, this finding did not survive multiple-testing correction, this polymorphism had previously been found to lack an association with schizophrenia as a whole [62], and no other published studies look at association with treatment-response.

The null result for Val66Met is somewhat surprising, as two larger studies have found robust associations when comparing clozapine users to non-clozapine users (but not specifically confirmed treatment-responders, as in this review) [63], and in a study looking at response to a single antipsychotic trial [64]. This may reflect a lack of power in the included study, or that the studies are capturing different phenotypes.

##### Reelin

Studies identified also suggest that treatment-resistance may be differentiated from treatment-responsive schizophrenia by greater repetition of the CCG repeat for the 5’UTR polymorphism on the reelin gene [34]. However, this was also not corrected for multiple-testing and this polymorphism had previously been found to lack an association with schizophrenia [65], despite reelin abnormalities having long been associated with schizophrenia [66]; unfortunately no other studies appear to look at an association with treatment response.

### Genetic loading

One study identified indicates that treatment-resistant schizophrenia may be differentiated from treatment-responsive schizophrenia by higher familial loading scores and greater risk of schizophrenia in relatives; in fact, the study found that treatment-responsive patients did not show the significantly higher risk compared to healthy volunteers that treatment-resistant patients did [39]. An earlier study looking at a single antipsychotic trial similarly found that first-degree relatives of non-responders to haloperidol had higher lifetime risk for schizophrenia spectrum disorders [67]. More recent studies have found associations between a history of clozapine treatment and a higher polygenic risk score [68] and treatment-resistance and higher genome-wide burden of rare duplications [69] (but neither compared to confirmed treatment-responders and were thus not included in this review). These cumulatively suggest that treatment-resistant schizophrenia may be more heritable, with stronger genetic influence.

### Immune system

One study identified indicates that treatment-resistance may be differentiated from treatment-responsive schizophrenia by predominance of the HLA-A1 allele [37] but another found null results for a difference in HLA antigen types [38].

Wider literature has indicated the importance of the immune system in treatment-response, with two studies finding that treatment-resistant patients show significantly higher levels of serum IL-6 levels than healthy volunteers (with non-resistant patients presenting with intermediate levels) [70, 71] (neither were included in this review because their non-resistant patients were not confirmed as treatment-responsive). Similarly, two GWAS’s investigating treatment response have also implicated variants associated with the immune system: one compared treatment-resistant patients to healthy volunteers and found an association with polymorphisms of SLAMF1, a gene associated with lymphocyte activation [72]; while another looked at response to a single antipsychotic trial and found an association with RTKN2, believed to be involved in lymphopoiesis [73].

### Neurocognitive function

Two papers identified indicate that treatment-resistant patients have greater deficits in neurocognitive domains, with a replicated result in verbal memory [42, 43]; this would be consistent with previous studies that have long reported associations between worsened memory and treatment-resistance [74]. However, cross-sectional studies suffer from symptom severity potentially confounding the results.

### Clinical variables

The one identified prospective study on PANSS scores reported that a lower score at baseline predicted treatment-resistance at 12 weeks, and found no association between improvement in the first 2 weeks of antipsychotic treatment and later treatment-response [40]. These are surprising findings, especially the latter as a 2014 review of treatment response (looking only at single-antipsychotic trials, and thus not included in the review) found that early lack of response was one of the most robust predictors of later lack of response [75], and a 2015 meta-analysis (again looking at only single-antipsychotic trials) found that lack of response at two weeks had high specificity and positive predictive validity for predicting later non-response [76]. It may be that early lack of response predicts later non-response for specific antipsychotics but not non-response in general (as characterised by treatment-resistance); however, this seems unlikely and it is much more plausible that the reported lack of association in the present review is due to the small sample size of the identified study.

One identified study indicated that treatment-resistance is associated with an earlier age of onset [41], consistent with multiple studies reporting that early age of onset is predictive of poor outcomes [4, 77] and a review by Kessler et al. [78] which reports that early age of onset is associated with more severe, persistent and treatment-refractory conditions across psychiatric disorders.

### Demographic characteristics

The one identified study indicates a greater proportion of white patients in treatment-resistant patient groups. This finding has been replicated by another study [79] (not included because it did not compare to confirmed treatment-responsive patients) but the implications of this are unclear. It has been fairly well-established that the excess of schizophrenia in black African populations typically seen is due to (as yet undetermined) non-genetic factors [80], so these findings suggest that treatment-resistant schizophrenia is less influenced by these environmental factors; this would be consistent with the above section suggesting increased heritability in the treatment-resistant patient group. Similarly, a large Danish epidemiological study [81] recently found that established environmental risk factors for schizophrenia such as urban environment did not predict treatment-resistance, and in fact were negatively correlated with treatment-resistance.

## Conclusions

Tentative evidence from a diverse range of research fields supports the idea that treatment-resistance may be a categorically distinct disorder; in contrast to treatment-responsive schizophrenia, treatment-resistant schizophrenia appears to be characterised by a relatively normal dopamine system but an abnormal glutamate system, and significant decreases in grey matter. There is some evidence indicating that treatment-resistant schizophrenia may be a broadly more genetic disorder, and differences may be partially driven by different genotypic variants, with explorative studies suggesting group differences in genes involved in neurotransmission, immune function and neural development; however, none of the gene-association findings are reported to survive multiple comparisons. Overall, there have been relatively few studies conducted comparing patients with strictly defined treatment-resistance and treatment-responsiveness, and there is little independent replication of significant findings. In addition, definitions of treatment-resistance and treatment-responsiveness remain inconsistent, and confounding variables have often not been systematically evaluated. Other reviews within the field highlight that variable populations, heterogeneous definitions, underpowered studies and the lack of prospective studies (particularly from illness-onset) create challenges for interpreting the literature [48].

Addressing the inconsistency of treatment-resistant definitions and the comparison groups used would be of great value for future research, especially when attempting to determine if there are categorical differences between these groups of patients. We recommend that the field develops and follows a consensus for more strictly defining and referring to treatment-resistance, incorporating the clinical guidelines as a minimum. This is underway with the Treatment Response and Resistance in Psychosis (TRRIP) Working Group, with guidelines in press [82]. In addition, studies should carefully decide their comparison groups and be very clear about whether it contains patients who are simply non-resistant or who are demonstrable treatment-responders; if the latter, they should be clear about their definition of treatment-responders, ideally aiming to use established consensus definitions for response (e.g., Andreasen remission criteria [83]).

Future studies should also seek to both further replicate the findings of glutamatergic and grey matter abnormalities, and advance knowledge in areas where there are contradictions or a simple lack of research. Investigating neurochemical differences between treatment-responsive and treatment-resistant patients may be of particular importance, as these could inform different approaches to pharmacological intervention. Neuroimaging studies may also use resting state data to investigate functional connectivity, as a recent study [84] and a review of the broader literature [48] have indicated this may have a role in predicting treatment response. In terms of genetic markers of treatment-resistant illness, psychiatric genetics as a field has moved away from the candidate-gene approach used in all genetic studies identified by the present review; future research should consider instead well-powered GWAS’s and more sophisticated models incorporating gene-environment interactions and epigenetic variation. Studies on social or environmental factors are also notably lacking in the literature. Across these fields, progressing from cross-sectional to prospective studies will be required to firmly determine whether observed differences are predictive of treatment response, or arise consequential to differing antipsychotic treatment regimens or duration of active symptoms. If future research can confirm treatment-resistant schizophrenia as a distinct disorder from treatment-responsive schizophrenia, a key next step towards personalised treatment will be then determining whether predictors can be used to identify treatment-resistance early in illness, and at an individual patient level.

## Additional files

Additional file 1: Appendix I: Search Strategies [Search terms used for databases]. Appendix II: Data Extraction Form [Form used to extract data from included studies]. Appendix III: Quality Assessment Tool [Adapted Newcastle-Ottawa Scale used for quality assessment of studies]. Appendix IV: Excluded Studies [Table listing all studies which underwent full-text screening and were subsequently excluded, with reasons for exclusion]. Appendix V: Included Studies [Table of all included studies with key details – study design, sample size, country, definitions of treatment-resistance and response, variables measured, full results, and overall quality assessment score]. Appendix VI: Quality Assessment for Included Studies [Tables outlining full quality assessment scoring for each included study]. (DOCX 180 kb)
Additional file 2: PRISMA CHECKLIST This additional file includes the PRISMA checklist for reporting systematic reviews; this has been completed for the current review, highlighting where in the review each item on the checklist is addressed. (DOCX 28 kb)

## Abbreviations

ACC: Anterior cingulate cortex
BDNF: Brain derived neurotrophic factor
DLPFC: Dorsolateral prefrontal cortex
EEG: Electroencephalography
Glx: Glutamate and glutamine
GWAS: Genome wide association study
HLA: Human leukocyte antigen
HVA: Homovanilic acid
MRI: Magnetic resonance imaging
MRS: Magnetic resonance spectroscopy
NAA: N-acetyl aspartate
PANSS: Positive and Negative Syndrome Scale (PANSS)
PET: Position emission tomography

## Footnotes

1 Demjaha et al. (2014) used a subset of patients reported in Demjaha et al. (2012). The 2012 paper reported on dopamine synthesis capacity, and the 2014 reported on dopamine synthesis capacity and levels of glutamate. Only the 2012 dopamine findings and 2014 glutamate findings are included in this review.

2 Molina et al. [29] and de Bartolomeis et al. [43] also measured PANSS scores but with cross-sectional designs
