# Supplementary material for: Is treatment-resistant schizophrenia categorically distinct from treatment-responsive schizophrenia? a systematic review
Source: BMC Psychiatry. 2017 Jan 13;17:12. doi: 10.1186/s12888-016-1177-y (PMC5237235; doi:10.1186/s12888-016-1177-y)
Supplement: Additional file 1: — Appendix I: Search Strategies [Search terms used for databases]. Appendix II: Data Extraction Form [Form used to extract data from included studies]. Appendix III: Quality Assessment Tool [Adapted Newcastle-Ottawa Scale used for quality assessment of studies]. Appendix IV: Excluded Studies [Table listing all studies which underwent full-text screening and were subsequently excluded, with reasons for exclusion]. Appendix V: Included Studies [Table of all included studies with key details – study design, sample size, country, definitions of treatment-resistance and response, variables measured, full results, and overall quality assessment score]. Appendix VI: Quality Assessment for Included Studies [Tables outlining full quality assessment scoring for each included study]. (DOCX 180 kb) [file 12888_2016_1177_MOESM1_ESM.docx]

**Appendix I - Search Strategies**

**Ovid**

- 1 schizophrenia.ab,kf,ti.
- 2 exp paranoid disorders/ or exp schizophrenia/
- 3 *Drug Resistance/
- 4 resistant.ab,kf,ti.
- 5 refractory.ab,kf,ti.
- 6 resistance.ab,kf,ti.
- 7 1 OR 2
- 8 3 OR 4 OR 5 OR 6
- 9 7 AND 8
- 10 limit to “all adults (19 plus years)”
- 11 limit to human/humans

**Pubmed**

Search **(((schizophrenia[Title/Abstract]) OR schizophrenia[MeSH Major Topic])) AND ((drug resistance[MeSH Major Topic]) OR (((resistance) OR resistant) OR refractory))**

Filters: **Humans**

**CINAHL**

- S1 - MJ schizophrenia OR TI schizophrenia OR AB schizophrenia
- S2 - MJ drug resistance OR AB resistance OR AB resistant OR AB refractory OR TI resistance OR TI refractory OR TI resistant
- S3 - S1 AND S2

**OpenGrey**

- drug resistance AND schizophrenia
- resistance AND schizophrenia
- refractory AND schizophrenia
- resistant AND schizophrenia

**Appendix II - Data Extraction Form**

| **Paper ID** |  |
| --- | --- |
| **First author** |  |
| **Year** |  |
| **Title** |  |
| **Contact Email** |  |
| **Journal** |  |
| **Volume** |  |
| **Page** |  |
| **Publication Type** |  |
| **Study Design** |  |
| **Study Duration and Recruitment Dates** |  |
| **Country** |  |
| **Clear Aims and Hypotheses** |  |
| **Sample Size** |  |
| **Power Calculation** |  |
| **Treatment-Resistant: Sample Size** |  |
| ***Treatment-Responsive: Sample Size*** |  |
| **Treatment-Resistant: Recruitment** |  |
| ***Treatment-Responsive: Recruitment*** |  |
| **Treatment-Resistant: Definition** |  |
| ***Treatment-Responsive: Definition*** |  |
| **Ascertainment of Diagnosis** |  |
| **Blinding** |  |
| **Treatment-Resistant: Age, Sex, Ethnicity, Age of Onset, etc.** |  |
| ***Treatment-Responsive: Age, Sex, Ethnicity, Age of Onset, etc.*** |  |
| **Drop out / Non-response / Missing Data** |  |
| **Predictors collected: -Definition -Unit of Measurement -Scale -Time of Measure -Ascertainment Method** |  |
| **All reported?** |  |
| **Primary Measure** |  |
| **Confounders -Design -Statistics -Discussion** |  |
| **Statistical Method -Including Grouping** |  |
| **Treatment-Resistant: Summary Data** |  |
| ***Treatment-Responsive: Summary Data*** |  |
| **Effect size, CI, p value** |  |
| **Key Conclusions** |  |
| **Other Comments from Author -Discussion of Limitations -Addressing Bias** |  |
| **Own Comments** |  |
| **Correspondence Required** |  |
| **Conflict of Interest / Funding Source** |  |

**Appendix III - Quality Assessment Tool**

**Newcastle-Ottawa Scale (adapted)**

This scale has been adapted from the Newcastle-Ottawa Quality Assessment Scale to perform a quality assessment of studies for the systematic review.

**SELECTION OF PARTICIPANTS (Maximum 5)**

**1) Ascertainment of treatment-resistance**

a) Independent assessment.*

b) Independent review of records.*

c) Record linkage.

d) Self-report.

e) No description.

**2) Ascertainment of treatment-responsiveness**

a) Confirmed response to antipsychotic treatment, with independent validation.*

b) Confirmed response to antipsychotic treatment, records or self-report.*

c) No mention of history.

**3) Selection of treatment-resistant patients**

a) All eligible cases with outcome of interest over a defined period of time, in a defined catchment area, or in a defined hospital or clinic, group of hospitals, health maintenance organization etc.*

b) An appropriate sample of those cases (e.g. random sample).*

c) Neither of the above, or not stated.

**4) Selection of treatment-responsive patients**

a) Derived from same population as treatment-resistant patients.*

b) Different source/different inclusion and exclusion.

c) No description.

**5) Sample size:**

a) Justified and satisfactory. *

b) Not justified.

**COMPARABILITY (Maximum 2)**

**Comparability on basis of design or analysis.**

Patient groups must be matched in the design and/or confounders must be adjusted for in the analysis. Statements of no differences between groups or that differences were not statistically significant are not sufficient for establishing comparability.

*(We have not selected one factor that is the most important for comparability, because the variables are not the same in each study. Thus, the principal factor should be identified for each study.)*

a) Study controls for _______________ .*

b) Study controls for any additional factor.*

**PREDICTOR (Maximum 4)**

**1) Ascertainment of predictor**

a) Validated measurement tool, blind to treatment-resistant/treatment-responsive status.**

b) Validated, objective measurement tool, not blind to treatment-resistant/treatment-responsive status.*

c) Written self-report or medical record only.

d) No description.

**2) Same method of ascertainment for treatment-resistant and treatment-responsive**

a) Yes.*

b) No.

**3) Statistical test:**

a) The statistical test used to analyse the data is clearly described and appropriate, and the measurement of the association is presented, and the probability level (p value).*

b) The statistical test is not appropriate, not described or incomplete.

| **Study (author, year)** | | | | | | | | |
| --- | --- | --- | --- | --- | --- | --- | --- | --- |
| **SELECTION (max 5)** | | | | | **COMPARABILITY (max 2)** | **PREDICTOR (max 4)** | | |
| **1) Ascertainment of treatment-resistant diagnosis** | **2) Ascertainment of treatment-responsive diagnosis** | **3)**  **Selection of treatment-resistant patients** | **4)**  **Selection of treatment-responsive patients** | **5)**  **Sample size** | **Comparability on basis of design or analysis.** | **1) Ascertainment of predictor** | **2)**  **Same method of ascertainment for treatment-resistance and treatment-responsiveness** | **3)**  **Statistical test** |
| a) Independent assessment.*  b) Independent review of records.*  c) Record linkage.  d) Self-report.  e) No description. | a) Confirmed response to antipsychotic treatment, with experimental validation.*  b) Confirmed response to antipsychotic treatment, interview, records or self-report.*  c) No description. | a) All eligible cases with outcome of interest over a defined period of time, in defined population etc.*  b) An appropriate sample of those cases (e.g. random sample).*  c) Neither of the above, or not stated. | a) Derived from same population as treatment-resistant.*  b) Different source/different inclusion and exclusion.  c) No / inadequate description. | a) Justified and satisfactory.*  b) Not justified. | a) Study controls for _____________.*  b) Study controls for any additional factor.* | a) Validated measurement tool, blind to treatment-resistant/treatment-responsive status.**  b) Validated, objective measurement tool, not blind to treatment-resistant/treatment-responsive status.*  c) Non-validated/ non-objective measurement tool, self-report or medical record only.  d) No description. | a) Yes.*  b) No. | a) The statistical test used to analyse the data is clearly described and appropriate, and the measurement of the association is presented, and the probability level (p value).*  b) The statistical test is not appropriate, not described or incomplete. |

**Selection:**

**Comparability:**

**Predictor:**

**Appendix IV - Excluded Studies**

| **Table 4.** Excluded Studies and Reasons | | |
| --- | --- | --- |
|  | **Reference** | **Reason for Exclusion** |
| 1 | Agid O, et al. Early prediction of clinical and functional outcome in schizophrenia. European Neuropsychopharmacology. 2013;23(8):842-851. | Doesn’t meet criteria for treatment-resistant definition |
| 2 | Bozina N, et al. Associations between MDR1 gene polymorphisms and schizophrenia and therapeutic response to olanzapine in female schizophrenic patients. Journal of Psychiatric Research. 2008;42(2):89-97. | Doesn’t meet criteria for treatment-resistant definition |
| 3 | Cohen RM et al. Abnormalities in the distributed network of sustained attention predict neuroleptic treatment response in schizophrenia. Neuropsychopharmacology. 1998;19(1):36-47. | Doesn’t meet criteria for treatment-resistant definition |
| 4 | Fijal BA, et al. Analysis of gene variants previously associated with iloperidone response in patients with schizophrenia who are treated with risperidone. Journal of Clinical Psychiatry. 2012;73(3):367-371. | Doesn’t meet criteria for treatment-resistant definition |
| 5 | Galinowski A, et al. Evolution of plasma homovanillic acid (HVA) in chronic schizophrenic patients treated with haloperidol. Acta Psychiatrica Scandinavica. 1998;97(6):458-466. | Doesn’t meet criteria for treatment-resistant definition |
| 6 | Hori A, et al. The biological background of refractory schizophrenia: A study on clinical states, serum drug concentration, cognitive function and brain CT findings. Japanese Journal of Psychiatry and Neurology. 1993;47(3):515-523. | Doesn’t meet criteria for treatment-resistant definition |
| 7 | Inada T, et al. Relationship between catechol-O-methyltransferase polymorphism and treatment-resistant schizophrenia. American Journal of Medical Genetics Neuropsychiatric Genetics. 2003;120(1):35-39. | Doesn’t meet criteria for treatment-resistant definition |
| 8 | Ji X, et al. An association between serotonin receptor 3B gene (HTR3B) and treatment-resistant schizophrenia (TRS) in a Japanese population. Nagoya Journal of Medical Science. 2008;70(1-2):11-17. | Doesn’t meet criteria for treatment-resistant definition |
| 9 | Ji X, et al. Relationship between three serotonin receptor subtypes (HTR3A, HTR2A and HTR4) and treatment-resistant schizophrenia in the Japanese population. Neuroscience Letters. 2008;435(2):95-98. | Doesn’t meet criteria for treatment-resistant definition |
| 10 | Kaymak SU, et al. Effects of duration of untreated psychosis on the clinical course and neurocognitive features of schizophrenia. Asia Pacific Psychiatry. 2012;4(1):40-47. | Doesn’t meet criteria for treatment-resistant definition |
| 11 | Kinon BJ, et al. Predicting response to atypical antipsychotics based on early response in the treatment of schizophrenia. Schizophrenia Research. 2008;102(1-3):230-240. | Doesn’t meet criteria for treatment-resistant definition |
| 12 | Kohlrausch FB, et al. Naturalistic pharmacogenetic study of treatment resistance to typical neuroleptics in European-Brazilian schizophrenics. Pharmacogenetics and Genomics. 2008;18(7):599-609. | Doesn’t meet criteria for treatment-resistant definition |
| 13 | Kolakowska T, et al. Drug-related and illness-related factors in the outcome of chlorpromazine treatment: Testing a model. Psychological Medicine. 1980;10(2):335-343. | Doesn’t meet criteria for treatment-resistant definition |
| 14 | Kondo T, et al. Combination of dopamine D2 receptor gene polymorphisms as a possible predictor of treatment-resistance to dopamine antagonists in schizophrenic patients. Progress in Neuro-Psychopharmacology & Biological Psychiatry. 2003;27(6):921-926. | Doesn’t meet criteria for treatment-resistant definition |
| 15 | Lawrie SM, et al. Magnetic resonance imaging and single photon emission tomography in treatment-responsive and treatment-resistant schizophrenia. British Journal of Psychiatry. 1995;167(2):202-210. | Doesn’t meet criteria for treatment-resistant definition |
| 16 | Lawrie SM, et al. Qualitative cerebral morpholgy in schizophrenia: A magnetic resonance imaging study and systematic literature review. Schizophrenia Research. 1997;25(2):155-166. | Doesn’t meet criteria for treatment-resistant definition |
| 17 | Lee SH, et al. Quantitative EEG and low resolution electromagnetic tomography (LORETA) imaging of patients with persistent auditory hallucinations. Schizophrenia Research. 2006;83(2-3):111-119. | Doesn’t meet criteria for treatment-resistant definition |
| 18 | Lysaker PH, et al. Deficits and predictors of improvement in schizophrenia. Journal of Nervous and Mental Disease. 1995;183(11):688-692. | Doesn’t meet criteria for treatment-resistant definition |
| 19 | Mauri MC, et al. Prediction of response to haloperidol in schizophrenia: Neuroendocrine, neuromorphological and clinical variables. International Clinical Psychopharmacology. 1994;9(1):3-7. | Doesn’t meet criteria for treatment-resistant definition |
| 20 | Medina-Hernandez V, et al. Increased lipid peroxidation and neuron specific enolase in treatment refractory schizophrenics. Journal of Psychiatric Research. 2007;41(8):652-658. | Doesn’t meet criteria for treatment-resistant definition |
| 21 | Mitelman SA, et al. Volume of the cingulate and outcome in schizophrenia. Schizophrenia Research. 2005;72(2-3):91-108. | Doesn’t meet criteria for treatment-resistant definition |
| 22 | Molina V, et al. Optimized voxel brain morphometry: association between brain volumes and the response to atypical antipsychotics. European Archives of Psychiatry & Clinical Neuroscience. 2011;261(6):407-416. | Doesn’t meet criteria for treatment-resistant definition |
| 23 | Nasrallah HA, et al. Cerebral ventricular enlargement and dopamine synthesis inhibition in chronic schizophrenia. Archives of General Psychiatry. 1980;37(12):1427. | Doesn’t meet criteria for treatment-resistant definition |
| 24 | Nimgaonkar VL, et al. 5-HT2 receptor gene locus: association with schizophrenia or treatment response not detected. Psychiatr Genet. 1996;6(1):23-27. | Doesn’t meet criteria for treatment-resistant definition |
| 25 | O'Gorman C, et al. Early improvement on antipsychotic treatment as a predictor of subsequent response in schizophrenia: analyses from ziprasidone clinical studies. Hum Psychopharmacol. 2011;26(4-5):282-290. | Doesn’t meet criteria for treatment-resistant definition |
| 26 | Ohara K, et al. (Anticipation and imprinting in schizophrenia. Biological Psychiatry. 1997;42(9):760-766. | Doesn’t meet criteria for treatment-resistant definition |
| 27 | Ota T, et al. Treatment resistant chronic psychopathology and CT scans in schizophrenia. Acta Psychiatrica Scandinavica. 1987;75(4):415-427. | Doesn’t meet criteria for treatment-resistant definition |
| 28 | Pae CU, et al. Interaction analysis between 5-HTTLPR and TNFA -238/-308 polymorphisms in schizophrenia. Journal of Neural Transmission. 2006;113(7):887-897. | Doesn’t meet criteria for treatment-resistant definition |
| 29 | Papazisis G, et al. Association of common ABCB1 polymorphisms with response to antipsychotic therapy in a naturalistic setting: Preliminary results. European Neuropsychopharmacology. 2014;24:S162. | Doesn’t meet criteria for treatment-resistant definition |
| 30 | Ramos J, et al. Abnormal EEG patterns in treatment-resistant schizophrenic patients. International Journal of Neuroscience. 2001;109(1-2):47-59. | Doesn’t meet criteria for treatment-resistant definition |
| 31 | Ramsey TL, et al. Evidence for a SULT4A1 haplotype correlating with baseline psychopathology and atypical antipsychotic response. Pharmacogenomics. 2011;12(4):471-480. | Doesn’t meet criteria for treatment-resistant definition |
| 32 | Roohafza H, et al. Lipid profile in antipsychotic drug users: A comparative study. ARYA Atherosclerosis. 2013;9(3):198-202. | Doesn’t meet criteria for treatment-resistant definition |
| 33 | Sajatovic M, et al. Clinical characteristics and health resource use of men and women veterans with serious mental illness. Psychiatric Services. 1997;48(11):1461-1463. | Doesn’t meet criteria for treatment-resistant definition |
| 34 | Sakalis G, et al. The possible role of metabolites in therapeutic response to chlorpromazine treatment. Psychopharmacologia. 1973;32(3):279-284. | Doesn’t meet criteria for treatment-resistant definition |
| 35 | Shaikh S, et al. Analysis of the conserved Asp(114) residue of the dopamine D2 receptor in schizophrenic patients. Psychiatr Genet. 1994;4(4):211-214. | Doesn’t meet criteria for treatment-resistant definition |
| 36 | Sinibaldi L, et al. Mutations of the Nogo-66 receptor (RTN4R) gene in schizophrenia. Human Mutation. 2004;24(6):534-535. | Doesn’t meet criteria for treatment-resistant definition |
| 37 | Smith RC, et al. Haloperidol: Plasma levels and prolactin response as predictors of clinical improvement in schizophrenia: Chemical v radioreceptor plasma level assays. Archives of General Psychiatry. 1984;41(11):1044-1049. | Doesn’t meet criteria for treatment-resistant definition |
| 38 | Smith GN, et al. Obstetric complications and severity of illness in schizophrenia. Schizophrenia Research. 1995;14(2):113-120. | Doesn’t meet criteria for treatment-resistant definition |
| 39 | Szulc A, et al. Proton magnetic resonance spectroscopy measures related to short-term symptomatic outcome in chronic schizophrenia. Neuroscience Letters. 2013;547:37-41. | Doesn’t meet criteria for treatment-resistant definition |
| 40 | van der Heijden FM, et al. Amino acids in schizophrenia: evidence for lower tryptophan availability during treatment with atypical antipsychotics?. Journal of Neural Transmission. 2005;112(4):577-585. | Doesn’t meet criteria for treatment-resistant definition |
| 41 | Wang L, et al. Response of risperidone treatment may be associated with polymorphisms of HTT gene in Chinese schizophrenia patients. Neuroscience Letters. 2007;414(1):1-4. | Doesn’t meet criteria for treatment-resistant definition |
| 42 | Watanabe Y, et al. Frontal lobe function and social adjustment in patients with schizophrenia: Near-infrared spectroscopy. Human Psychopharmacology. 2015;30(1):28-41. | Doesn’t meet criteria for treatment-resistant definition |
| 43 | Xu HD, et al. Anticipation and imprinting in schizophrenia. Biological Psychiatry. 1997;42(9):760-766. | Doesn’t meet criteria for treatment-resistant definition |
| 44 | Yasui-Furukori N, et al. Association between multidrug resistance 1 (MDR1) gene polymorphisms and therapeutic response to bromperidol in schizophrenic patients: a preliminary study. Progress in Neuro-Psychopharmacology & Biological Psychiatry. 2006;30(2):286-291. | Doesn’t meet criteria for treatment-resistant definition |
| 45 | Yue W, et al. Association of DAOA polymorphisms with schizophrenia and clinical symptoms or therapeutic effects. Neuroscience Letters. 2007;416(1):96-100. | Doesn’t meet criteria for treatment-resistant definition |
| 46 | Yu WY, et al. Short telomeres in patients with chronic schizophrenia who show a poor response to treatment. Journal of Psychiatry and Neuroscience. 2008;33(3):244-247. | Doesn’t meet criteria for treatment-resistant definition |
| 47 | Zlotowski M. Behavioral differences between process and reactive schizophrenics in a monotonous repetitive task. Journal of Personality and Social Psychology. 1965;1(3):240-244. | Doesn’t meet criteria for treatment-resistant definition |
| 48 | Aitchison KJ, et al. Failure to respond to treatment with typical antipsychotics is not associated with CYP2D6 ultrarapid hydroxylation. British Journal of Clinical Pharmacology. 1999;48(3):388-394. | Doesn’t meet criteria for treatment-responsive definition |
| 49 | Alonso-Solis A, et al. Default mode network alterations in refractory schizophrenia patients with auditory verbal hallucinations. European Neuropsychopharmacology. 2013;23:S267-S268. | Doesn’t meet criteria for treatment-responsive definition |
| 50 | Bechter K, et al. Cerebrospinal fluid analysis in affective and schizophrenic spectrum disorders: Identification of subgroups with immune responses and blood-CSF barrier dysfunction. Journal of Psychiatric Research. 2010;44(5):321-330. | Doesn’t meet criteria for treatment-responsive definition |
| 51 | Bilic P, et al. Treatment-resistant schizophrenia and DAT and SERT polymorphisms. Gene. 2014;543(1):125-132. | Doesn’t meet criteria for treatment-responsive definition |
| 52 | Bourque J, et al. Clozapine and visuospatial processing in treatment-resistant schizophrenia. Cognitive Neuropsychiatry. 2013;18(6):615-630. | Doesn’t meet criteria for treatment-responsive definition |
| 53 | Brunstein MG, et al. Increased serum adenosine deaminase activity in schizophrenic receiving antipsychotic treatment. Neuroscience Letters. 2007;414(1):61-64. | Doesn’t meet criteria for treatment-responsive definition |
| 54 | Caspi A, et al. Premorbid behavioral and intellectual functioning in schizophrenia patients with poor response to treatment with antipsychotic drugs. Schizophrenia Research. 2007;94(1-3):45-49. | Doesn’t meet criteria for treatment-responsive definition |
| 55 | Cezaretto M., et al. (2014). "Clinical and sociodemographic profile of patients with refractory schizophrenia treated in a tertiary center. Jornal Brasileiro de Psiquiatria 63(3): 185-190. | Doesn’t meet criteria for treatment-responsive definition |
| 56 | Ciobanu A. Schizophrenia with obsessive-compulsive disorder comorbidity: Clinical and treatment particularities. European Archives of Psychiatry and Clinical Neuroscience. 2013;1:S79. | Doesn’t meet criteria for treatment-responsive definition |
| 57 | Findling RL, et al. Premorbid asociality in neuroleptic-resistant and neuroleptic-responsive schizophrenia. Psychological Medicine. 1996;26(5):1033-1041. | Doesn’t meet criteria for treatment-responsive definition |
| 58 | Gadelha A, et al. Plasma Ndel1 enzyme activity is reduced in patients with schizophrenia - A potential biomarker?. Journal of Psychiatric Research. 2013;47(5):657-663. | Doesn’t meet criteria for treatment-responsive definition |
| 59 | Haussleiter IS, et al. NVE. Schizophrenia Research. 2012;140(1-3):258-259. | Doesn’t meet criteria for treatment-responsive definition |
| 60 | Hons J, et al. Different serine and glycine metabolism in patients with schizophrenia receiving clozapine. Journal of Psychiatric Research. 2012;46(6):811-818. | Doesn’t meet criteria for treatment-responsive definition |
| 61 | Hotta Y, et al. Association study between Disrupted-in-Schizophrenia-1 (DISC1) and Japanese patients with treatment-resistant schizophrenia (TRS). Progress in Neuro Psychopharmacology & Biological Psychiatry. 2011;35(2):636-639. | Doesn’t meet criteria for treatment-responsive definition |
| 62 | Jia P, et al. Association of FAS, a TNF-alpha receptor gene, with treatment resistant schizophrenia. Schizophrenia Research. 2011;129(2-3):211-212. | Doesn’t meet criteria for treatment-responsive definition |
| 63 | Kubera KM, et al. Source-based morphometry of gray matter volume in patients with schizophrenia who have persistent auditory verbal hallucinations. Progress in Neuro Psychopharmacology and Biological Psychiatry. 2014;50:102-109. | Doesn’t meet criteria for treatment-responsive definition |
| 64 | La YJ, et al. Decreased levels of apolipoprotein A-I in plasma of schizophrenic patients. Journal of Neural Transmission. 2007;114(5):657-663. | Doesn’t meet criteria for treatment-responsive definition |
| 65 | Lee M, et al. Decreased plasma tryptophan and tryptophan/large neutral amino acid ratio in patients with neuroleptic-resistant schizophrenia: Relationship to plasma cortisol concentration. Psychiatry Research. 2011;185(3):328-333. | Doesn’t meet criteria for treatment-responsive definition |
| 66 | Lin A, et al. The inflammatory response system in treatment-resistant schizophrenia: Increased serum interleukin-6. Schizophrenia Research. 1998;32(1):9-15. | Doesn’t meet criteria for treatment-responsive definition |
| 67 | Malherbe PJ, et al. Phenotypic features of patients with schizophrenia carrying de novo gene mutations: A pilot study. Psychiatry Research. 2015;225(1-2):108-114. | Doesn’t meet criteria for treatment-responsive definition |
| 68 | Monteleone P, et al. Prolactin hyperresponsiveness to {d}-fenflumarine in drug-free schizophrenic patients: A placebo-controlled study. Biological Psychiatry. 1999;45(12):1606-1611. | Doesn’t meet criteria for treatment-responsive definition |
| 69 | Mortimer AM, et al. Clozapine for treatment-resistant schizophrenia: National Institute of Clinical Excellence (NICE) guidance in the real world. Clinical Schizophrenia & Related Psychoses. 2010;4(1):49-55. | Doesn’t meet criteria for treatment-responsive definition |
| 70 | Mouaffak F, et al. Ultra-resistant schizophrenia is not associated with the multidrug-resistant transporter 1 (MDR1) gene rs1045642 variant. Journal of Clinical Psychopharmacology. 2011;31(2):236-238. | Doesn’t meet criteria for treatment-responsive definition |
| 71 | Mundo E, et al. MCP-1 gene (SCYA2) and schizophrenia: A case-control association study. American Journal of Medical Genetics Neuropsychiatric Genetics. 2005;132(1):1-4. | Doesn’t meet criteria for treatment-responsive definition |
| 72 | Noto C, et al. Circulating levels of sTNFR1 as a marker of severe clinical course in schizophrenia. Journal of Psychiatric Research. 2013;47(4):467-471. | Doesn’t meet criteria for treatment-responsive definition |
| 73 | Ortiz BB, et al. Is disorganized schizophrenia a predictor of treatment resistance? Evidence from an observational study. Revista Brasileira de Psiquiatria. 2013;35(4) 432-434. | Doesn’t meet criteria for treatment-responsive definition |
| 74 | Ota VK, et al. DRD1 rs4532 polymorphism: A potential pharmacogenomic marker for treatment response to antipsychotic drugs. Schizophrenia Research. 2012;142(1-3):206-208. | Doesn’t meet criteria for treatment-responsive definition |
| 75 | Ota VK, et al. Polymorphisms in schizophrenia candidate gene UFD1L may contribute to cognitive deficits. Psychiatry Research. 2013;209 (1):110-113. | Doesn’t meet criteria for treatment-responsive definition |
| 76 | Ota VK, et al. PRODH Polymorphisms, Cortical Volumes and Thickness in Schizophrenia. PloS one. 2014;9(2):e87686. | Doesn’t meet criteria for treatment-responsive definition |
| 77 | Roberts RC, et al. Dopaminergic synapses in the caudate of subjects with schizophrenia: Relationship to treatment response. Synapse. 2009;63(6):520-530. | Doesn’t meet criteria for treatment-responsive definition |
| 78 | Schmitt A, et al. Increased serum interleukin-1 and interleukin-6 in elderly, chronic schizophrenic patients on stable antipsychotic medication. Neuropsychiatric Disease and Treatment. 2005;1(2):171-177. | Doesn’t meet criteria for treatment-responsive definition |
| 79 | Somerville SM, et al. Mitochondria in the striatum of subjects with schizophrenia: Relationship to treatment response. Synapse. 2010;65(3):215-224. | Doesn’t meet criteria for treatment-responsive definition |
| 80 | Sorensen HJ, et al. An association between autumn birth and clozapine treatment in patients with schizophrenia: a population-based analysis. Nordic Journal of Psychiatry. 2014;68(6):428-432. | Doesn’t meet criteria for treatment-responsive definition |
| 81 | Spivak B, et al. The impact of clozapine treatment on serum lipids in chronic schizophrenic patients. Clinical Neuropharmacology. 1999;22(2):98-101. | Doesn’t meet criteria for treatment-responsive definition |
| 82 | Stroup TS, et al. Geographic and clinical variation in Clozapine use in the United States. Psychiatric Services. 2014;65(2):186-192. | Doesn’t meet criteria for treatment-responsive definition |
| 83 | Sumiyoshi T, et al. Sex differences in plasma homovanillic acid levels in schizophrenia and normal controls: Relation to neuroleptic resistance. Biological Psychiatry. 1997;41(5):560-566. | Doesn’t meet criteria for treatment-responsive definition |
| 84 | Takao T, et al. Association of treatment-resistant schizophrenia with the G2677A/T and C3435T polymorphisms in the ATP-binding cassette subfamily B member 1 gene. Psychiatric Genetics. 2006;16(2):47-48. | Doesn’t meet criteria for treatment-responsive definition |
| 85 | Takebayashi Y, et al. No genetic association between SLC7A10 and Japanese patients with schizophrenia. Progress in Neuro Psychopharmacology and Biological Psychiatry. 2011;35(8):1965-1968. | Doesn’t meet criteria for treatment-responsive definition |
| 86 | Teo C, et al. Analysis of treatment-resistant schizophrenia and 384 markers from candidate genes. Pharmacogenetics and Genomics. 2012;22(11):807-811. | Doesn’t meet criteria for treatment-responsive definition |
| 87 | Teo C, et al. The role of ethnicity in treatment refractory schizophrenia. Comprehensive Psychiatry. 2013;54(2):167-172. | Doesn’t meet criteria for treatment-responsive definition |
| 88 | Vrtunski PB, et al. Effect of clozapine on motor function in schizophrenic patients. Schizophrenia Research. 1996;20(1-2):187-198. | Doesn’t meet criteria for treatment-responsive definition |
| 89 | Wheeler AJ. Treatment pathway and patterns of clozapine prescribing for schizophrenia in New Zealand. Annals of Pharmacotherapy. 2008;42(6):852-860. | Doesn’t meet criteria for treatment-responsive definition |
| 90 | Wimberley T, et al. Treatment-resistant schizophrenia and clozapine treatment: A population-based pharmacoepidemiological study. European Journal of Epidemiology. 2013;1:S119. | Doesn’t meet criteria for treatment-responsive definition |
| 91 | Wolf ND, et al. Magnetic resonance perfusion imaging of auditory verbal hallucinations in patients with schizophrenia. Schizophrenia Research. 2012;134(2-3):285-287. | Doesn’t meet criteria for treatment-responsive definition |
| 92 | Xiang YT, et al. Clozapine use in schizophrenia: Findings of the Research on Asia Psychotropic Prescription (REAP) studies from 2001 to 2009. Australian and New Zealand Journal of Psychiatry. 2011;45(11):968-975. | Doesn’t meet criteria for treatment-responsive definition |
| 93 | Zhang JP, et al. Genetic variation in BDNF is associated with antipsychotic treatment resistance in patients with schizophrenia. Schizophrenia Research. 2013;146(1-3):285-288. | Doesn’t meet criteria for treatment-responsive definition |
| 94 | Zugman A, et al. Reduced dorso-lateral prefrontal cortex in treatment resistant schizophrenia. Schizophrenia Research. 2013;148(1-3):81-86. | Doesn’t meet criteria for treatment-responsive definition |
| 95 | Maes M, et al. Effects of atypical antipsychotics on the inflammatory response system in schizophrenic patients resistant to treatment with typical neuroleptics. European Neuropsychopharmacology. 2000;10(2):119-124. | Doesn’t meet criteria for treatment-responsive definition |
| 96 | Maes M, et al. Increased serum interleukin-8 and interleukin-10 in schizophrenic patients resistant to treatment with neuroleptics and the stimulatory effects of clozapine on serum leukemia inhibitory factor receptor. Schizophrenia Research. 2002;54(3):281-291. | Doesn’t meet criteria for treatment-responsive definition |
| 97 | Mouaffak F, et al. Association of an UCP4 (SLC25A27) haplotype with ultra-resistant schizophrenia. Pharmacogenomics. 2011;12(2):185-193. | Doesn’t meet criteria for treatment-responsive definition |
| 98 | Mouaffak F, et al. Association of Disrupted in Schizophrenia 1 (DISC1) missense variants with ultra-resistant schizophrenia. Pharmacogenomics Journal. 2011;11(4):267-273. | Doesn’t meet criteria for treatment-responsive definition |
| 99 | Ritsner M, et al. Cortisol/dehydroepiandrosterone ratio and responses to antipsychotic treatment in schizophrenia. Neuropsychopharmacology. 2005;30(10):1913-1922. | Doesn’t meet criteria for treatment-responsive definition |
| 100 | Martin A, et al. Clozapine use in a forensic population in a New South Wales prison hospital. Australian & New Zealand Journal of Psychiatry. 2008;42(2):141-146. | Doesn’t meet criteria for treatment-responsive definition |
| 101 | Molina V, et al. Increase in gray matter and decrease in white matter volumes in the cortex during treatment with atypical neuroleptics in schizophrenia. Schizophrenia Research. 2005;80(1):61-71. | Comparison to neuroleptic-naive |
| 102 | Molina V, et al. Marked hypofrontality in clozapine-responsive patients. Pharmacopsychiatry. 2007;40(4):157-162. | Comparison to neuroleptic-naive |
| 103 | Lin CH, et al. No evidence for association of serotonin-2A receptor variant (102T/C) with schizophrenia or clozapine response in a Chinese population. NeuroReport. 1999;10(1):57-60. | Comparison to healthy |
| 104 | Machado de Sousa JP & Hallak JE. Neurocognitive functioning and facial affect recognition in treatment-resistant schizophrenia treated with clozapine. Schizophrenia Research. 2008;106(2-3):371-372. | Comparison to healthy |
| 105 | Nolan KA & Krakowski M. Psychopathology and aggression in patients with treatment-resistant schizophrenia. Psychiatric Times. 2006;23(12):13. | Comparison to healthy |
| 106 | Angelopoulos E, et al. Cortical interactions during the experience of auditory verbal hallucinations. The Journal of Neuropsychiatry and Clinical Neurosciences. 2011;23(3):287-293. | Treatment-resistance not measured |
| 107 | Bigliani V, et al. In vivo occupancy of striatal and temporal cortical D2/D3 dopamine receptors by typical antipsychotic drugs. The British Journal of Psychiatry. 1999;175:231-138. | Treatment-resistance not measured |
| 108 | Bredski J, et al. The prediction of discharge from in-patient psychiatric rehabilitation: A case-control study. BMC Psychiatry. 2011;11:149. | Treatment-resistance not measured |
| 109 | Brunelin J, et al. Impaired verbal source monitoring in schizophrenia: An intermediate trait vulnerability marker?. Schizophrenia Research. 2007;89(1-3):287-292. | Treatment-resistance not measured |
| 110 | Cooper SJ, et al. Aggressive behavior in a pyschiatric observation ward. Acta Psychiatrica Scandinavica. 1983;68(5):386-393. | Treatment-resistance not measured |
| 111 | Dapsys K, et al. ECT and information processing in patients with treatment-resistant psychiatric disorders assessed by event-related potential p300. European Psychiatry. 2013;28. | Treatment-resistance not measured |
| 112 | Dean B. [3H]RX 821002 in human dorsolateral prefrontal cortex: no changes in postmortem tissue from subjects with schizophrenia. Psychiatry Research. 2003;119(1-2):25-31. | Treatment-resistance not measured |
| 113 | Hassan S, et al. Trait perfectionism and perfectionistic self-presentation in early psychosis. Early intervention in psychiatry. 2012;6:58. | Treatment-resistance not measured |
| 114 | Kendler KS, et al. Gender and schizophrenia. Results of an epidemiologically-based family study. British Journal of Psychiatry. 1995;167(2):184-192. | Treatment-resistance not measured |
| 115 | Lee SH, et al. Quantitative EEG and low resolution electromagnetic tomography (LORETA) imaging of patients with persistent auditory hallucinations. Schizophrenia Research. 2006;83(2-3):111-119. | Treatment-resistance not measured |
| 116 | Lee SH, et al. Nonlinear analysis of electroencephalogram in schizophrenia patients with persistent auditory hallucination. Psychiatry Investigation. 2008;5(2):115-120. | Treatment-resistance not measured |
| 117 | Magaro PA. An hypothesis concerning the relations between drive and size estimation for paranoids and nonparanoids within specific schizophrenic subgroups. Perceptual and Motor Skills. 1970;31(2):489-490. | Treatment-resistance not measured |
| 118 | Malaspina D, et al. Relation of familial schizophrenia to negative symptoms but not to the deficit syndrome. The American Journal of Psychiatry. 2000;157(6):994-1003. | Treatment-resistance not measured |
| 119 | Marinescu I, et al. Choroid plexus calcification: clinical, neuroimaging and histopathological correlations in schizophrenia. Romanian Journal of Morphology & Embryology. 2013;54(2):365-369. | Treatment-resistance not measured |
| 120 | McEvoy JP, et al. Insight in schizophrenia. Its relationship to acute psychopathology. Journal of Nervous & Mental Disease. 1989;177(1):43-47. | Treatment-resistance not measured |
| 121 | Meagher DJ, et al. Longitudinal assessment of psychopathological domains over late-stage schizophrenia in relation to duration of initially untreated psychosis: 3-year prospective study in a long-term inpatient population. Psychiatry Research. 2004;126(3):217-227. | Treatment-resistance not measured |
| 122 | Pandurangi AK, et al. Relation of serum molindone levels to serum prolactin levels and antipsychotic response. Journal of Clinical Psychiatry. 1989;50(10):379-381. | Treatment-resistance not measured |
| 123 | Picci RL, et al. Does substance use disorder affect clinical expression in first-hospitalization patients with schizophrenia? Analysis of a prospective cohort. Psychiatry Research. 2013;210(3):780-786. | Treatment-resistance not measured |
| 124 | Tauscher J, et al. Equivalent occupancy of dopamine D1 and D2 receptors with clozapine: Differentiation from other atypical antipsychotics. The American Journal of Psychiatry. 2004;161(9):1620-1625. | Treatment-resistance not measured |
| 125 | Tovilla-Zarate CA, et al. Association study between the MDR1 gene and clinical characteristics in schizophrenia. Revista Brasileira de Psiquiatria. 2014;36(3):227-232. | Treatment-resistance not measured |
| 126 | Verdoux H & Bourgeois M. Obstetrical complications and schizophrenia. Comparative study of obstetric antecedents in schizophrenic and bipolar patients. Encephale. 1993;19(4):313-320. | Treatment-resistance not measured |
| 127 | Braverman ER. Brain electrical activity mapping in treatment resistant schizophrenics. Journal of Orthomolecular Medicine. 1990;5(1):46-48. | No comparison |
| 128 | Buckley PF, et al. Clinical and biochemical correlates of "high-dose" clozapine therapy for treatment-refractory schizophrenia. Schizophrenia Research. 2001;49(1-2):225-227. | All patients treatment-resistant |
| 129 | Kelly DL, et al. Nonresponse to clozapine and premorbid functioning in treatment refractory schizophrenia. Comprehensive Psychiatry. 2010;51(3):298-302. | All patients treatment-resistant |
| 130 | Moresco RM, et al. Cerebral D2 and 5-HT2 receptor occupancy in schizophrenic patients treated with olanzapine or clozapine. Journal of Psychopharmacology. 2004;18(3):355-365. | All patients treatment-resistant |
| 131 | Moroji T, et al. A neuroendocrinological study of responders and non-responders to ceruletide treatment in chronic neuroleptic-resistant schizophrenia. Progress in Neuro Psychopharmacology & Biological Psychiatry. 1987;11(1):49-64. | All patients treatment-resistant |
| 132 | Nair CJ, et al. Does akathisia influence psychopathology in psychotic patients treated with clozapine?. Biological Psychiatry. 1999;45(10):1376-1383. | All patients treatment-resistant |
| 133 | Volonteri LS, et al. Long-acting injectable risperidone and metabolic ratio: A possible index of clinical outcome in treatment-resistant schizophrenic patients. Psychopharmacology. 2010;210(4):489-497. | All patients treatment-resistant |
| 134 | Angelopoulos E, et al. Cortical interactions during the experience of auditory verbal hallucinations. Journal of Neuropsychiatry & Clinical Neurosciences. 2011;23(3):287-293. | Symptom mechanism |
| 135 | Fukuzako H, et al. Phosphorus magnetic resonance spectroscopy in schizophrenia: correlation between membrane phospholipid metabolism in the temporal lobe and positive symptoms. Progress in Neuro Psychopharmacology & Biological Psychiatry. 1996;20(4):629-640. | Symptom mechanism. |
| 136 | Dean B. [H]RX 821002 in human dorsolateral prefrontal cortex: No changes in postmortem tissue from subjects with schizophrenia. Psychiatry Research. 2003;119(1-2):25-31. | Drug mechanism |
| 137 | Daskalakis ZJ & George TP. Clozapine, GABA B, and the treatment of resistant schizophrenia. Clinical Pharmacology and Therapeutics. 2009;86(4):442-446. | Drug mechanism |
| 138 | Weintraub P & Wamboldt MZ. Expressed emotion in child psychiatry: A risk factor for psychopathology and treatment resistance?. Current Opinion in Psychiatry. 1996;9(4):241-246. | Not specific to SCZ |
| 139 | Agius M & Zdanowicz N. Editorial: On neuroscience, epidemiology, pharmacotherapy, recovery, treatment resistance, and community mental health teams. Psychiatria Danubina. 2011;23(1):S5-S7. | Not original research |
| 140 | Bartzokis G & Altshuler L. Biological underpinnings of treatment resistance in schizophrenia: an hypothesis. Psychopharmacology Bulletin. 2003;37(4):5-7. | Not original research |
| 141 | Boutros NN. P300 amplitude reduction in schizophrenia: an endophenotype or an illness indicator?. Clinical Neurophysiology. 2008;119(12):2669-2670. | Not original research |
| 142 | Buckley PF. Is It Treatment-Refractory Schizophrenia...And if It Is?. Psychiatric Times. 2011;28(6):24-24. | Not original research |
| 143 | Doi N & Usui C. Are high-risk haplotypes in DTNBP1 and NRG1 resistance genes for schizophrenia?. American Journal of Psychiatry. 2006;163(5):940-941. | Not original research |
| 144 | Elkis H. Clozapine, refractory schizophrenia and evidences. Revista Brasileira de Psiquiatria. 2001;23(2):59-60. | Not original research |
| 145 | Hemmingsen R, et al. Cortical brain dysfunction in early schizophrenia: Secondary pathogenetic hierarchy of neuroplasticity, psychopathology and social impairment. Acta Psychiatrica Scandinavica Supplementum. 1999;99(395):80-88. | Not original research |
| 146 | Johnstone EC & Sandler R. Treatment resistance in schizophrenia. BMJ. 1996;312(7027):325-326. | Not original research |
| 147 | Manji HK, et al. Bromocriptine in neuroleptic resistance. Biological Psychiatry. 1987;22(4):523-524. | Not original research |
| 148 | Sumiyoshi T. Neurobiological aspects of schizophrenia. Journal of the California Alliance for the Mentally Ill. 1996;7(4):53-54. | Not original research |
| 149 | Managing the Lifecycle of Schizophrenia: First Episode, Stabilised and Treatment-Resistant Patients: Introduction. European Neuropsychopharmacology. 1996;6(2). | Not original research |
| 150 | Buckley PF. Persistent symptoms in treatment-resistant schizophrenia. Johns Hopkins Advanced Studies in Medicine. 2005;5(7B):S757-762. | TRS N = 1 |
| 151 | Paus S, et al. Acute hebephrenia. Archives of Neurology. 2005;62(8):1312-1313. | TRS N = 1 |
| 152 | Bani-Fatemi A, et al. Potential and direct methylation of 5-HT receptors in treatment resistant schizophrenia. European Neuropsychopharmacology. 2014;24:S562. | Potentially eligible but unable to confirm with author |
| 153 | Barksdale KA, et al. Synaptic proteins in the postmortem anterior cingulate cortex in schizophrenia: Relationship to treatment and treatment response. Neuropsychopharmacology. 2014;39(9):2095-2103. | Potentially eligible but unable to confirm with author |
| 154 | Gadelha A, et al. Plasma Ndel1 enzyme activity is reduced in patients with schizophrenia - A potential biomarker?. Journal of Psychiatric Research. 2013;47(5):657-663. | Potentially eligible but unable to confirm with author |
| 155 | Gilbert E, et al. Cluster analysis of cognitive deficits may mark heterogeneity in schizophrenia in terms of outcome and response to treatment. European Archives of Psychiatry & Clinical Neuroscience. 2014;264(4):333-343. | Potentially eligible but unable to confirm with author |
| 156 | Kecmanovic M, et al. Schizophrenia and apolipoprotein E gene polymorphism in Serbian population. International Journal of Neuroscience. 2010;120(7):502-506. | Potentially eligible but unable to confirm with author |
| 157 | Li J & Meltzer HY. A genetic locus in 7p12.2 associated with treatment resistant schizophrenia. Schizophrenia Research. 2014;159(2-3):333-339. | Potentially eligible but unable to confirm with author |
| 158 | Quarantelli M, et al. Patients with poor response to antipsychotics have a more severe pattern of frontal atrophy: a voxel-based morphometry study of treatment resistance in schizophrenia. BioMed Research International. 2014;325052-325052. | Potentially eligible but unable to confirm with author |
| 159 | Roberts RC, et al. Synaptophysin, vGLUT1, mitofusin2 and calcineurin protein levels in the anterior cingulate cortex in schizophrenia: Relation to treatment and treatment response. Neuropsychopharmacology. 2013;38:S128-S129. | Potentially eligible but unable to confirm with author |
| 160 | Uhlhaas PJ, et al. Theory of mind and perceptual context-processing in schizophrenia. Cognitive Neuropsychiatry. 2006;11(4):416-436. | Potentially eligible but unable to confirm with author |
| 161 | Acuna M, et al. Influence of negative symptoms in schizophrenia therapeutic response: A critical study. Actas Luso-Espanolas de Neurologia, Psiquiatria y Ciencias Afines. 1998;26(4):209-213. | Unable to access full text version / necessary details |
| 162 | Brown WA, et al. The 24-hour dexamethasone suppression test in a clinical setting: relationship to diagnosis, symptoms, and response to treatment. American Journal of Psychiatry. 1979;136(4b):543-547. | Unable to access full text version / necessary details |
| 163 | Ciganik I, et al. Experience with radiotherapy in therapeutically resistant schizophrenia. Ceskoslovenska psychiatrie. 1985;81(3):176-180. | Unable to access full text version / necessary details |
| 164 | Dikii NT. Predicting the effect of psychopharmacotherapy in schizophrenic patients. Zhurnal Nevropatologii i Psikhiatrii Imeni S - S – Korsakova. 1982;82(1):110-115. | Unable to access full text version / necessary details |
| 165 | Durand D & Caceda R. Comparison of clinical characteristics and outcomes of patients on clozapine and long acting antipsychotics. Clinical Pharmacology and Therapeutics. 2012;91:S92. | Unable to access full text version / necessary details |
| 166 | Escande M, et al. The negative subtypes of schizophrenia. Psychologie Medicale. 1994;26(10):1044-1048. | Unable to access full text version / necessary details |
| 167 | Gamburg A, et al. Neurohormonal regulation of the lability of myoneural synapses in schizophrenics. Zhurnal Nevropatologii i Psikhiatrii. 1969;69(5):711-718. | Unable to access full text version / necessary details |
| 168 | Gonzalez JC, et al. Persistent auditory hallucinations. Psychopathology. 2006;39(3):120-125. | Unable to access full text version / necessary details |
| 169 | Govorin NV. Immunogenetic HLA markers in predicting therapeutic resistance in paranoid schizophrenia. Zhurnal Nevropatologii i Psikhiatrii imeni S S Korsakova. 1991;91(3):90-93. | Unable to access full text version / necessary details |
| 170 | Govorin NV, et al. Significance of disorders of the processes of lipid peroxidation in patients with persistent paranoid schizophrenia resistant to the treatment. Zhurnal Nevropatologii i Psikhiatrii Imeni S - S – Korsakova. 1991;91(7):121-124. | Unable to access full text version / necessary details |
| 171 | Govorin NV & Lozhkina AN. Antibodies to neuroleptics and their role in the mechanisms of the development of drug resistance in psychopharmacotherapy of patients with paranoid schizophrenia. Zhurnal Nevropatologii i Psikhiatrii Imeni S - S – Korsakova. 1991;91(7):117-121. | Unable to access full text version / necessary details |
| 172 | Holden JM, et al. Echoencephalographic patterns in chronic schizophrenia (relationship to therapy resistance). Biological Psychiatry. 1973;6(2):129-141. | Unable to access full text version / necessary details |
| 173 | Holden JM, et al. Predictor patterns in the management of therapy-resistant schizophrenia. Dis Nerv Syst. 1971;32(4):260-268. | Unable to access full text version / necessary details |
| 174 | Kinon BJ, et al. Possible predictors of neuroleptic-resistant schizophrenic relapse: Influence of negative symptoms and acute extrapyramidal side effects. Psychopharmacology Bulletin. 1993;29(3):365-369. | Unable to access full text version / necessary details |
| 175 | Kinon BJ, et al. Treatment of neuroleptic-resistant schizophrenic relapse. Psychopharmacology Bulletin. 1993;29(2):309-314. | Unable to access full text version / necessary details |
| 176 | Kuznetsova NI, et al. Anti-phenothiazine antibodies and C-reactive protein as possible indicators of developing therapeutic resistance to phenothiazines. Laboratornoe delo. 1979;(7):419-422. | Unable to access full text version / necessary details |
| 177 | Lee MA, et al. Effects of clozapine in cognitive function in schizophrenia. Journal of Clinical Psychiatry. 1994;55(B):82-87. | Unable to access full text version / necessary details |
| 178 | Leszek J, et al. Natural human leukocyte interferon in the treatment of schizophrenia. The European Journal of Psychiatry. 1991;5(1):55-63. | Unable to access full text version / necessary details |
| 179 | Lieberman JA, et al. The development of treatment resistance in patients with schizophrenia: A clinical and pathophysiologic perspective. Journal of Clinical Psychopharmacology. 1998;18(2,1):20S-24S. | Unable to access full text version / necessary details |
| 180 | Missaoui S, et al. Early-onset schizophrenia: Clinical and endophenotypical features. Annales Medico-Psychologiques. 2008;166(8):633-637. | Unable to access full text version / necessary details |
| 181 | Perez Castejon MJ, et al. Regional cerebral perfusion and response to clozapine in refractory schizophrenia. Revista Espanola de Medicina Nuclear. 1996;15(5):317-324. | Unable to access full text version / necessary details |
| 182 | Pu CC, et al. Clinical features of patients with treatment-resistant schizophrenia. Chinese Mental Health Journal. 2012;26(8):566-570. | Unable to access full text version / necessary details |
| 183 | Ramos J, et al. Impairments in attention and facial emotion recognition in treatment refractory and non refractory schizophrenics evaluated through an odd-ball paradigm. Revista de Neurologia. 2001;33(11):1027-1032. | Unable to access full text version / necessary details |
| 184 | Reine G, et al. Clinical experience of using clozapine. Psychologie Medicale. 1992;24(14):1583-1595. | Unable to access full text version / necessary details |
| 185 | Saito M & Kita N. Clinical approach to therapy-resistant schizophrenia by quantitative EEG analysis. Seishin Shinkeigaku Zasshi - Psychiatria et Neurologia Japonica. 1983;85(9):600-606. | Unable to access full text version / necessary details |
| 186 | Sanchez P, et al. Attention deficits and response to drug therapy in patients with treatment-resistant schizophrenia: Results through confirmatory factor analysis. Revista de Psiquiatria y Salud Mental. 2010;3(2):40-49. | Unable to access full text version / necessary details |
| 187 | Sharma T & Kerwin R. Biological determinants of difficult to treat patients with schizophrenia. British Journal of Psychiatry. 1996;Supplementum(31):5-9. | Unable to access full text version / necessary details |
| 188 | Sokolski KN, et al. Effects of substance abuse on hallucination rates and treatment responses in chronic psychiatric patients. Journal of Clinical Psychiatry. 1994;55(9):380-387. | Unable to access full text version / necessary details |
| 189 | Stassen H, et al. Predicting response to psychopharmacological treatment: Survey of recent results. Pharmacopsychiatry. 2011;44(6):263-272. | Unable to access full text version / necessary details |
| 190 | Terada T, et al. Profiles of clinical symptoms of refractory schizophrenia and of the pharmacotherapy for them. Seishin Igaku (Clinical Psychiatry). 1994;36(12):1245-1250. | Unable to access full text version / necessary details |
| 191 | Terzic T, et al. Treatment resistant schizophrenia in Slovenian population. Zdravniski Vestnik. 2014;83(9):573-580. | Unable to access full text version / necessary details |
| 192 | Thampi A, et al. Eye movements and neurocognitive function in treatment resistant schizophrenia: A pilot study. Irish Journal of Psychological Medicine. 2003;20(1):6-10. | Unable to access full text version / necessary details |
| 193 | Yang KJ, et al. Analysis of factors related to poor curative effect of early-on-set schizophrenia. Chinese Journal of Clinical Rehabilitation. 2005;9(44):89-91. | Unable to access full text version / necessary details |
| 194 | Boston PF, et al. Serum cholesterol and treatment-resistance in schizophrenia. Biological Psychiatry. 1996;40(6):542-543. | Score of low quality in quality assessment measure |
| 195 | Grodzicki J, et al. Differences in [3H]-Spiperone binding to peripheral blood lymphocytes from neuroleptic responsive and nonresponsive schizophrenic patients. Biological Psychiatry. 1990;27:1327-1330. | Score of low quality in quality assessment measure |
| 196 | Molina V, et al. Subcortical and cortical gray matter differences between Kraepelinian and non-Kraepelinian schizophrenia patients identified using voxel-based morphometry. Psychiatry Research: Neuroimaging. 2010;184(1):16-22. | Score of low quality in quality assessment measure |
| 197 | Neto JH, & Elkis H. Clinical aspects of super-refractory schizophrenia: A 6-month cohort observational study. Revista de Psiquiatria do Rio Grande do Sul. 2007;29:228-232. | Score of low quality in quality assessment measure |
| 198 | Itil TM, et al. Computerized EEG: predictor of outcome in schizophrenia. The Journal of nervous and mental disease. 1975;160(3),188-203. | No statistics |

**Appendix V - Included Studies**

| **Table 5.** Included Studies | | | | | | | |
| --- | --- | --- | --- | --- | --- | --- | --- |
| **Reference** | **First author (year)**  **Title** | **Study design**    **Total sample size (treatment-resistant, treatment-responsive)**  **Country** | **Definition of treatment-resistance** | **Definition of treatment-response** | **Variables measured** | **Results**  **AR = treatment-responsive (antipsychotic responders)**  **TR = treatment-resistant**  **UTR = ultra-treatment resistant** | **Quality Assessment** |
| 28 | Anderson (2015)  “Extensive Gray Matter Volume Reduction in Treatment-Resistant Schizophrenia” | Cross-sectional  37 (19, 18)  New Zealand | Treatment resistance was defined as a lack of significant symptom improvement following at least two trials of different antipsychotic agents at therapeutic doses for a minimum of 6 weeks each. All were on clozapine.  This study also included 15 ultra-treatment resistant (UTR) patents. These patients were put on alternative or additional  antipsychotics because their symptoms did not respond to clozapine  alone. | On first line antipsychotics. good treatment responders - *a 50% Brief Psychiatric Rating Scale (BPRS)/Positive and Negative Syndrome Scale (PANSS) reduction, which translates to a score of 2 on Clinical Global Impression-Global Improvement (i.e., CGI-I, much improved)** | Whole brain, grey matter, and white matter tissue volumes (cm^3^) | **Whole brain volume**  **Mean (SD)**  *AR:* 1560.8 (66.0)  *TR:* 1530.1 (68.9)  *UTR:* 1501.7 (55.7)  Mean differences  NS  **Gray matter volume**  *AR:* 817.3 (46.6)  *TR:* 781.5 (38.5)  *UTR:* 764.1 (27.5)  Mean difference  AR > TR 35.9 (17.3–54.4) p < 0.001  AR > UTR 53.2 (33.3-73.1) p < 0.001  **White matter volume**  *AR:* 743.5 (37.7)  *TR:* 748.7 (42.7)  *UTR:* 737.5 (38.1)  Mean differences  NS  **Peripheral cortex gray matter volume**  *AR:* 664.9 (40.8)  *TR:* 628.5 (35.1)  *UTR:* 620.2 (23.8)  Mean differences  AR > TR 36.4 (20.5–52.3, p < 0.001)  AR > UTR 44.7 (27.7-61.7, p < 0.001)  **Sub-analysis**  **VBM** Areas showing significantly less GM in TR patients compared with ARs  **Inferior temporal gyrus (Right)**  p < 0.001  **Post-central gyrus (Bilateral)**  p < 0.006  **Middle frontal gyrus (Left)**  p < 0.007  **Anterior supramarginal gyrus (Right)**  p < 0.010  **Superior frontal gyrus (Bilateral)**  p < 0.006  **Superior temporal gyrus (temporal pole) (Right)**  p < 0.036  **Lateral occipital cortex (Right)**  p < 0.028  **Supplementary motor cortex (Left)**  p < 0.039 | Selection: ***  Comparability: *  Predictor: ***  7 MODERATE |
| 29 | Molina (2008)  “Differential clinical, structural and P300 parameters in schizophrenia patients resistant to conventional neuroleptics” | Cross-sectional, with follow-up  49 (30, 19)  Spain | Persistence of clinically relevant positive symptoms (at least one positive symptom in the PANSS scoring 5 or more) and a CGI score equal to or higher than 4, despite the use at adequate doses of two different classical antipsychotics for a period longer than 2 months each. | Similar illness duration to TRS but had responded to haloperidol in the last year. | Cerebral tissue volumes, longitudinal volume changes over 6 months of atypical antipsychotics (clozapine in TR, olanzapine in AR), and P300 amplitudes | **Baseline tissue volumes**  Raw (SD), Residuals (SD)  **GM**  ***Frontal***  *AR:* 136.74 (19.2), -8.70 (11.8)  *TR:* 121.20 (19.6), -20.08 (14.2)  ***Parietal***  *AR:* 109.28 (14.2), -14.51 (10.0)  *TR:* 101.74 (13.1), -8.77 (15.5)  ***Occipital***  *AR:* 64.15 (8.9), -2.76 (6.6)  *TR:* 56.33 (7.7), -8.59 (7.7)  ***Temporal***  *AR:* 140.64 (13.7), -2.63 (6.4)  *TR:* 131.9 (12.4), -6.67 (9.9)  **WM**  ***Frontal***  *AR:* 102.09 (13.0), -2.81 (10.6)  *TR:* 109.83 (15.0), 9.77 (11.7)  ***Parietal***  *AR:* 107.39 (13.2), -.76 (9.5)  *TR:* 116.73 (15.9), 13.50 (11.6)  ***Occipital***  *AR:* 45.98 (6.6), .95 (5.0)  *TR:* 50.6 (57.0), 8.56 (5.6)  ***Temporal***  *AR:* 66.24 (6.8), 1.28 (5.0)  *TR:* 65.56 (8.7), 3.10 (6.4)  **ICV**  *AR:* 1450 (1410)  *TR:* 1392.75 (135.1)  All NS  **Stepwise discriminant analysis**  Occipital WM residuals have the strongest predictive value (Wilk's λ=0,641, χ2=21.12, p~.0001). 76.7% of the TR patients and 85.0% of the non-TR patients were classified correctly.  **P300 μV**  Mean (SD)  *AR:* 8.37 (5.2) (n=13)  *TR:* 5.46 (5.8) (n=11)  NS | Selection: ***  Comparability: *  Predictor: ***  7 MODERATE |
| 17 | Demjaha (2012)  “Dopamine Synthesis Capacity in Patients With Treatment-Resistant Schizophrenia” | Cross-sectional (PET)  24 (12, 12)  UK | Modified Kane criteria : 1. Drug-refractory condition: At least two prior drug trials of 4- to 6-weeks duration at 400 to 600 mg of chlorpromazine (or equivalent) with no clinical improvement. 2. Persistence of illness: > 5 years with no period of good social or occupational functioning. 3. Persistent psychotic symptoms: BPRS total score > 45 (on 18-item scale) and item score > 4 (moderate) on at least two of four positive symptom items. | Met remission in schizophrenia working group criteria for treatment remission: a score of mild or less (Positive and Negative Syndrome Scale item scores of ≤3; BPRS item scores of ≤3, using the 1–7 range for each item; SAPS and SANS item scores of ≤2) simultaneously on all items, a period of 6 months as a minimum time threshold during which the aforementioned symptom severity must be maintained. Individuals may remain in remission while experiencing minor changes in symptoms, in the absence of appreciable effects on daily function or subjective well-being. | Striatal volume, presynaptic striatal dopamine (whole striatum, and associative, limbic and sensorimotor subdivision) | **Striatal volumes mm^3^**  Mean (SD)  ***Whole Striatum***  *AR:* 16,424.1 (1,818.9)  *TR:* 15,590.9 (1,675.2)  NS  ***Associative***  *AR:* 10,185.0 (1,164.1) *TR:* 9,644.0 (1,165.6)  NS  ***Limbic***  *AR:* 1,965.5 (348.0) *TR:* 1,953.1 (262.3)  NS  ***Sensorimotor***  *AR:* 4,189.6 (513.5) *TR:* 4,260.2 (928.6)  NS  **Mean presynaptic striatal dopamine values (K_i_^cer^)**  ***Whole Striatum***  *AR:* 1.4 x 10^-2^ (0.18 x 10^-2^)  *TR*: 1.3 x 10^-2^ (0.14 x 10^-2^)  p0.02 corrected, effect size 1.11  ***Associative Subdivisions***  *AR:* 1.4 x 10^-2^ (0.15 x 10^-2^)  *TR:* 1.2 x 10^-2^ (0.14 x 10^-2^)  p0.008 corrected, effect size 1.31  ***Limbic Subdivisions***  *AR:* 1.5 x 10^-2^ (0.23 x 10^-2^)  *TR:* 1.3 x 10^-2^ (0.17 x 10^-2^)  p0.03 corrected, effect size 1.04  ***Sensorimotor Subdivisions***  *AR*: 1.6 x 10^-2^ (0.24 x 10^-2^)  *TR*: 1.4 x 10^-2^ (0.2 x 10^-2^)  NS  **Voxel-based Analysis**  Peak in head of caudate – greater values in AR to TR, p0.039. | Selection: ***  Comparability: **  Predictor: ***  8 MODERATE |
| 18 | Demjaha (2014)  Correspondence  “Antipsychotic Treatment Resistance in Schizophrenia Associated with Elevated Glutamate Levels but Normal Dopamine Function” | Cross-sectional (PET and MRS)  14 (6, 8)  UK | Modified Kane criteria: 1. Drug-refractory condition: At least two prior drug trials of 4- to 6-weeks duration at 400 to 600 mg of chlorpromazine (or equivalent) with no clinical improvement. 2. Persistence of illness: > 5 years with no period of good social or occupational functioning. 3. Persistent psychotic symptoms: BPRS total score > 45 (on 18-item scale) and item score > 4 (moderate) on at least two of four positive symptom items. | Met remission in schizophrenia working group criteria for treatment remission: a score of mild or less (Positive and Negative Syndrome Scale item scores of ≤3; BPRS item scores of ≤3, using the 1–7 range for each item; SAPS and SANS item scores of ≤2) simultaneously on all items, a period of 6 months as a minimum time threshold during which the aforementioned symptom severity must be maintained. Individuals may remain in remission while experiencing minor changes in symptoms, in the absence of appreciable effects on daily function or subjective well-being. | Anterior cingulate cortex metabolite concentrations (glutamate, Glx, NAA, choline, creatine, myo-inositol) | **Anterior cingulate cortex metabolite concentrations**  ***Glutamate***  *AR:* 8.87 (2.44)  *TR:* 10.32 (1.41)  t12 = 1.3 p=0.22 NS  ***Glx***  *AR:* 13.33 (2.85)  *TR:* 13.76 (2.86)  NS  ***NAA***  *AR:* 7.48 (1.21)  *TR:* 9.77 (0.87)  p=0.02  ***Choline***  *AR:* 2.15 (0.55)  *TR:* 2.41 (0.48)  NS  ***Creatine***  *AR:* 7.25 (1.37)  *TR:* 8.44 (0.83)  NS  ***Myo*-*inositol***  *AR:* 6.48 (1.64)  *TR:* 7.42 (1.69)  NS | Selection: **  Comparability: **  Predictor: ***  7 MODERATE |
| 27 | Goldstein (2015)  Glutamatergic neurometabolites in clozapine-responsive  and resistant schizophrenia | Case-control  31 (16,15)  New Zealand | Treatment resistant patients taking clozapine monotherapy prescribed in accordance with criteria for TRS using published algorithms (NICE, APA), and at most mildly ill (CGI)  This study also included 11 ultra-treatment resistant (UTR) patents. These patients were on a combination of antipsychotics having failed a trial of clozapine monotherapy (not including cessation due to side-effects). | Taking i) a second generation (atypical) non-clozapine antipsychotic (first-line responders), and at most mildly ill (CGI)  - *a 50% Brief Psychiatric Rating Scale (BPRS)/Positive and Negative Syndrome Scale (PANSS) reduction, which translates to a score of 2 on Clinical Global Impression-Global Improvement (i.e., CGI-I, much improved)** | Glutamatergic metabolites (Glx/Cr, NAA/Cr, Glu/Cr, Cho/Cr) in DLPFC, ACC and Putman | Mean (SD)  **DLPFC**  ***Glx/Cr***  *AR:* 1.50 (0.06)  *TR:* 1.37 (0.06)  *UTR:* 1.24 (0.07)  AR > UTRS MD = 0.25, SE = 0.09, p = 0.04  ***NAA/Cr***  *AR:* 1.46 (0.07)  *TR:* 1.51 (0.06)  *UTR:* 1.60 (0.07)  NS  ***Glu/Cr***  *AR:* 1.23 (0.05)  *TR:* 1.17 (0.05)  *UTR:* 1.08 (0.06)  NS  ***Cho/Cr***  *AR:* 0.34 (0.01)  *TR*: 0.35 (0.01)  *UTR*: 0.37 (0.01)  NS  **ACC**  ***Glx/Cr***  *AR*: 2.31 (0.16)  *TR*: 2.12 (0.16)  *UTR*: 2.28 (0.19)  NS  ***NAA/Cr***  *AR*: 1.63 (0.08)  *TR*: 1.58 (0.08)  *UTR*: 1.72 (0.010)  NS  ***Glu/Cr***  *AR*: 1.92 (0.11)  *TR*: 1.78 (0.11)  *UTR*: 1.91 (0.13)  NS  ***Cho/Cr***  *AR:* 0.36 (0.01)  *TR:* 0.37 (0.01)  *UTR:* 0.38 (0.02)  NS  **Putamen**  ***Glx/Cr***  *AR:* 1.53 (0.08)  *TR:* 1.83 (0.10)  *UTR:* 1.43 (0.10)  TR > AR MD = 0.31, SE = 0.12, p = 0.05  TR > UTR MD = 0.39, SE = 0.12, p = 0.02  ***NAA/Cr***  *AR*: 1.06 (0.19)  *TR*: 1.02 (0.24)  *UTR*: 1.22 (0.16)  NS  ***Glu/Cr***  *AR*: 1.12 (0.13)  *TR*: 1.23 (0.19)  *UTR*: 1.08 (0.17)  NS  ***Cho/Cr***  *AR*: 0.26 (0.01)  *TR*: 0.25 (0.01)  *UTR*: 0.27 (0.01)  NS | Selection: ***  Comparability: **  Predictor: ***  8 MODERATE |
| 30 | Anttila (2005)  “Lack of association between two polymorphisms of brain-derived neurotrophic factor and response to typical neuroleptics” | Gene-association  94 (51,43)  Finland | Failed to respond to treatment with at least two different typical antipsychotics. The minimum treatment period had to be 4 weeks and the minimum dose for each treatment period 400 mg chlorpromazine equivalent and at least 2 different standard neuroleptic drugs had to be used. The treatment periods occurred in hospital settings. The severity of schizophrenic symptoms had to be at least 4 on Clinical Global Impression scale. In addition, at least one of the following symptoms had to be present: conceptual disorganization, suspiciousness, hallucinatory behavior, and unusual thought content. | Sufficient and long-lasting response to typical antipsychotics. Before initiation of neuroleptic treatment, the severity of schizophrenic symptoms had to be at least 4 according to the Clinical Global Impression scale. | G169A (val66met) and C270T polymorphism of BDNF gene | **Distribution of the BDNF G196A and C270T polymorphisms**  **%**  **G196A**  ***GG***  *AR*: 69.8  *TR*: 68.6  ***GA***  *AR*: 27.9  *TR*: 27.5  ***AA***  *AR*: 2.3  *TR*: 3.9  **C270T**  ***CC***  *AR*: 83.7  *TR*: 84.3  ***CT***  *AR*: 16.3  *TR*: 11.8  ***TT***  *AR*: 0  *TR*: 3.9  AR vs TR, p>0.3 NS  **Sub-group analyses**  **Heterozygous vs. homozygous**  NS  **A allele carriers vs. those not carrying A allele (G196A polymorphism)**  NS  **T allele carriers vs. those not carrying that allele (C270T polymorphism)**  NS  **Haplotype analysis of the BDNF polymorphisms**  ***GC***  *AR*: 76.4  *TR*: 73.5  ***AC***  *AR*: 15.4  *TR*: 16.7  ***GT***  *AR*: 7.3  *TR*: 8.9  ***AT***  *AR*: 0.9  *TR*: 0.9  OR=0.842 (95% CI 0.43–1.64), p=0.612 NS | Selection: ****  Comparability: -  Predictor: ***  7 MODERATE |
| 33 | Anttila (2007)  “Association between 5-HT2A, TPH1 and GNB3 genotypes and response to typical neuroleptics: a serotonergic approach” | Gene-association  94 (51, 43)  Finland | Patients on clozapine medication who had failed to respond to treatment with two different conventional antipsychotics on at least two different occasions in a hospital setting.  In each index treatment period with conventional antipsychotics the lowest accepted daily dose was 400 mg chlorpromazine equivalent for a minimum of four weeks. Prior to the initiation of clozapine treatment the severity of symptoms of schizophrenia had to be ≥ 4 on the CGI Scale, and at least one of the following symptoms had to be present: conceptual disorganization, suspiciousness, hallucinatory behavior, or unusual thought content | Experienced sufficient and sustained response to treatment with typical antipsychotics: both the patient's psychotic symptoms had disappeared and the level of functioning had returned to a level corresponding to that prior the psychotic episode. Before initiation of antipsychotic treatment, the severity of schizophrenia symptoms had to be ≥ 4 according to the Clinical Global Impression Scale (CGI). | 5-HT2A T102C polymorphism, TPH1 779 polymorphism and GNB3 C825T polymorphism | **Genotype distribution %**  **5HT2A**  ***CC***  *AR*: 39.5 (F: 34.6, M: 47.1)  *TR*: 54.0 (F: 76.2, M: 37.9)  Female, OR = 6.04 (95% Cl 1.67–21.93), p = 0.005    ***CT***  *AR:* 53.5 (F: 53.8, M: 52.9)  *TR:* 38.0 (F: 19.0, M: 51.7)  NS  ***TT***  *AR*: 7.0 (F: 11.5, M: 0)  *TR*: 8.0% (F: 4.8, M: 10.3)  NS  **TPH1**  ***CC***  *AR:* 32.6 (F: 30.8, M: 35.3)  *TR*: 23.5 (F: 13.6, M: 31.0)  ***CA***  *AR:* 32.6 (F: 34.6, M: 29.4)  *TR:* 54.9 (F: 59.1, M: 51.7)  OR = 0.59 (95% Cl 0.36–0.98), p = 0.030  ***AA***  *AR*: 34.8 (F: 34.6, M: 35.3)  *TR*: 21.6 (F: 27.3, M: 17.3)  NS  **GNB3**  ***CC***  *AR:* 41.9 (F: 53.9, M: 23.5)  *TR:* 53.0 (F: 59.1, M: 48.3)  NS  ***CT***  *AR:* 46.5 (F: 26.9, M: 76.5)  *TR:* 43.1 (F: 36.4, M: 48.3)  Male OR = 3.48 (95% Cl 0.92–13.25), p = 0.061  ***TT***  *AR*: 11.6 (F: 19.2, M: 0)  *TR*: 3.9 (f 4.5, 3.4)  NS | Selection: ***  Comparability: -  Predictor: ****  7 MODERATE |
| 34 | Goldberger (2005)  “Population-based and family-based association study of 5'UTR polymorphism of the reelin gene and schizophrenia” | Gene-association  193 (45, 148)  France | No clinical remission despite several trials with different antipsychotic drugs for a sufficient duration and still requiring permanent day care, score 5–7 on May Dencker score. | At least one partial clinical remission allowing their discharge from hospital (score 1–4) | (CGG) repeat polymorphism of reelin gene | **CGG allele distribution**  **%**  **4**  *AR:* 0.3  *TR:* 0  **8**  *AR:* 43.9  *TR:* 33.3  **9**  *AR:* 0.3  *TR*: 1.1  **10**  *AR:* 48  *TR:* 62.2  X2 = 5.60, p = 0.03  **11**  *AR:* 1.3  *TR:* 1.1  **12**  *AR:* 2.3  *TR:* 0  **13**  *AR:* 3.3  *TR:* 2.2  **14**  *AR*: 0.3  *TR*: 0  **Distribution of Genotypes**  **4/10**  *AR*: 0.7  *TR*: 0  **8/8**  *AR*: 18.2  *TR*: 8.9  **8/10**  *AR*: 41.2  *TR*: 48.9  **8/11**  *AR*: 2  *TR*: 0  **8/12**  *AR*: 4.1  *TR*: 0  **8/13**  *AR*: 4.1  *TR*: 0  **9/10**  *AR*: 0.7  *TR*: 2.2  **10/10**  *AR*: 25  *TR*: 33.3  **10/11**  *AR*: 0.7  *TR*: 2.2  **10/13**  *AR*: 2  *TR*: 4.4  **10/14**  *AR*: 0.7  *TR*: 0  **12/13**  *AR*: 0.7  *TR*: 0  **Sub-Analysis**  (CGG)_10_ containing genotypes  AR vs TR, 91.1% vs 70.9%, X2 = 7.6, df=1,p = 0.006  Odds ratio for treatment-non response for (CCG)_10_  OR = 4.2, CI = 1.4;12.4  May Dencker score of response, patients with vs without the (CGG)_10_ allele  *With* 3.9 (0.9)  *Without* 3.6 (0.7)  F1.191=5.7, p=0.02) | Selection: ***  Comparability: -  Predictor: ***  6 MODERATE |
| 32 | Joober (1999)  “T102C polymorphism in the 5HT2A gene and schizophrenia: relation to phenotype and drug response variability” | Gene-association  102 (63, 39)  Canada | Continuous psychotic symptoms with no significant remission within the past 2 years, at least 3 periods of treatment with typical neuroleptics at optimal clinical requirements with no significant relief of symptoms in the preceding 5 years, and the inability to function with-out supervision in all or nearly all domains of social and vocational activities within the last 12 months. | Had at least 1 admission to a psychiatric care facility because of an acute psychotic episode. They always experienced full or partial remission in response to treatment with typical neuroleptics and were able to function with only occasional supervision in all or nearly all domains of social and vocational activities. They were required to be in remission or quasi-remission when compliant with their medication. | T102C polymorphism in the 5HT2A gene | **T102C genotypes %**  **1/1**  *AR*: 17.9 (F: 27.3, M: 14.3)  *TR*: 11.1 (F: 6.2, M: 12.5)  **1/2**  *AR*: 43.6 (F: 45.4, M: 42.8)  *TR*: 38.1 (F: 50.0, M: 34.0)  **2/2**  *AR*: 38.5 (F: 27.3, M: 42.8)  *TR*: 50.8 (F: 43.7, M: 53.2)  All NS  **No of alleles %**  **1**  *AR*: 39.7 (F: 50.0, M: 35.7)  *TR*: 30.2 (F: 31.2, M: 29.7)  **2**  *AR*: 60.2 (F: 50.0, M: 64.9)  *TR*: 69.8 (F: 68.75, M: 70.2)  All NS | Selection: ***  Comparability: -  Predictor: ****  7 MODERATE |
| 35 | Kampman (2006)  “RGS4 genotype is not associated with antipsychotic medication response in schizophrenia” | Gene-association  93 (50, 43)  Finland | Patients on clozapine medication who had failed to respond to treatment with two different conventional antipsychotics on at least two different occasions in a hospital setting.  In each index treatment period with conventional antipsychotics the lowest accepted daily dose was 400 mg chlorpromazine equivalent for a minimum of four weeks. Prior to the initiation of clozapine treatment the severity of symptoms of schizophrenia had to be ≥ 4 on the CGI Scale, and at least one of the following symptoms had to be present: conceptual disorganization, suspiciousness, hallucinatory behaviour, or unusual thought content | Experienced sufficient and sustained response to treatment with typical antipsychotics: both the patient's psychotic symptoms had disappeared and the level of functioning had returned to a level corresponding to that prior the psychotic episode. Before initiation of antipsychotic treatment, the severity of schizophrenia symptoms had to be ≥ 4 according to the Clinical Global Impression Scale (CGI). | Rs 951436 polymorphism on RGS4 gene | **RGS4 allele distributions %**  **G allele**  *AR*: 50  *TR*: 47  **T allele**  *AR*: 50  *TR*: 53  p0.68 NS | Selection: ***  Comparability: -  Predictor ***  6 MODERATE |
| 36 | Krebs (1998)  “Dopamine D3 receptor gene variants and substance abuse in schizophrenia” | Gene-association  89 (19, 70)  France | Scale by May et al. A score of at least 5 (no clinical remission despite several trials with different neuroleptics for a sufficient duration and necessity for permanent day care). | "At least partly responded to neuroleptics" | Bal I polymorphism of DRD3 allele | **Allele 1 %**  *AR*: 63.6  *TR*: 65.8  NS  **Genotype distribution %**  **1-1**  *AR*: 42.9  *TR*: 31.6  **2-2**  *AR*: 15.7  *TR*: 0  **1-2**  *AR*: 41.4  *TR*: 68.4  X2 = 5.76, d.f. = 1, P = 0,016  **Frequency of homozygosity %**  **1-1 + 2-2**  *AR*: 58.6  *TR*: 31.6  X2 = 4.37, d.f. = 1, P = 0.037 | Selection: **  Comparability: -  Predictor: ****  6 MODERATE |
| 31 | Krebs (2000)  “Brain Derived Neurotrophic Factor (BDNF) gene variants -association with age at onset and therapeutic response in schizophrenia“ | Gene-association  88 (20, 68)  France | No clinical remission despite several trials with different antipsychotic drugs for a sufficient duration and required permanent day care. | "At least a partial clinical remission under antipsychotic treatment allowing discharge from hospital" | Dinucleotide repeat polymorphism of BDNF gene | **BDNF allele distribution %**  **176bp**  *AR*: 0.74  *TR*: 0  **174bp (A1)**  *AR*: 27.9  *TR*: 15  **172bp (A2)**  *AR*: 3.7  *TR*: 0  **170bp (A3)**  *AR*: 62.5  *TR*: 70  **168bp (A4)**  *AR*: 4.4  *TR*: 15  **166bp (A5)**  *AR*: 0.74  *TR*: 0  WI = 2106; P = 0.01  **Sub-analysis**  Frequency of long alleles (172–176 bp)  X2 = 4.6, P = 0.04, OR = 2.7. | Selection: **  Comparability: -  Predictor: ****  6 MODERATE |
| 37 | Lahdelma (1998)  “Association between HLA-A1 allele and schizophrenia gene(s) in patients refractory to conventional neuroleptics but responsive to clozapine medication” | Gene-association  38 (19,19)  Finland | Kane criteria (used BPRS). Responsive to Clozapine. | 20% decrease in the BPRS (Brief Psychiatric Rating Scale) total scale plus either a post-treatment Clinical Global Impressions (CGI) scale rating of mildly ill (≤3) or a post-treatment PBRS score of 35 or less – in response to conventional neuroleptics* | HLA allele type | **HLA-A1 %**  **A1**  *AR*: 10.5  *TR*: 57.9  Difference 37.9%, P=0.0011 uncorrected (7.2-68.6%), p=0.064 corrected  **A2**  *AR*: 57.9  *TR*: 47.4  **A3**  *AR*: 57.9  *TR*: 42.1  **A9**  *AR*: 5.3  *TR*: 5.3  **A10**  *AR*: 15.8  *TR*: 5.3  **A11**  *AR*: 5.3  *TR*: 5.3  **A19**  *AR*: 10.5  *TR*: 15.8  **A28**  *AR*: 15.8  *TR*: 5.3  **B5**  *AR*: 15.8  *TR*: 15.8  **B7**  *AR*: 42.1  *TR*: 21.1  **B8**  *AR*: 10.5  *TR*: 31.6  **B12**  *AR*: 0  *TR*: 10.5  **B13**  *AR*: 0  *TR*: 5.3  **B15**  *AR*: 31.6  *TR*: 15.8  **B16**  *AR*: 10.5  *TR*: 10.5  **B18**  *AR*: 5.3  *TR*: 10.5  **B22**  *AR*: 5.3  *TR*: 5.3  **B27**  *AR*: 15.8  *TR*: 15.8  **B35**  *AR*: 26.3  *TR*: 26.3  **B40**  *AR*: 10.5  *TR*: 0  **DR1**  *AR*: 57.9  *TR*: 52.6  **DR2**  *AR*: 31.6  *TR*: 21.1  **DR3**  *AR*: 26.3  *TR*: 26.3  **DR4**  *AR*: 26.3  *TR*: 21.1  **DR5**  *AR*: 5.3  *TR*: 5.3  **DR6**  *AR*: 26.3  *TR*: 26.3  **DR7**  *AR*: 5.3  *TR*: 10.5  **DR8**  *AR*: 10.5  *TR*: 5.3  **DR9**  *AR*: 0  *TR*: 10.5  **HLA-A1 + B8 haplotypes (frequency)**  *AR*: 0.022  *TR*: 0.157  P=0.025. | Selection: ****  Comparability: -  Predictor: ***  7 MODERATE |
| 38 | Meged (1999)  “Human leukocyte antigen typing, response to neuroleptics and clozapine-induced agranulocytosis in Jewish Israeli schizophrenic patients” | Cross-sectional  88 (50, 38)  Israel | Absence of response to at least three neuroleptic agents from different chemical classes. If two phenothiazines were administered, they had to be of different subclasses (either aliphatic, piperdine or piperazine side chains). Dosage of each medication equivalent to or greater than chlopromazine 1000mg daily and duration of treatment at least 8 weeks.  All TRS were put on clozapine and after 12 weeks response was determined. 25/50 TRS patients showed no improvement in response to clozapine, and therefore may be considered equivalent to UTRS. | All previously responsive to standard neuroleptics. During study patients given haloperidol to max dose of 30mg/day for 12 weeks. Patients who didn't improve were switched. Criteria of treatment response in both groups were a sufficient improvement for discharge to a residential or outpatient setting, or a CGI rating of "much improved" or "very much improved" after 12 weeks. | HLA antigen frequency | **HLA antigens (% of patients)**  **HLA A26**  *AR*: 5.26  *TR*: 16  *UTR*: 20  NS  **HLA B36**  *AR*: 18.42  *TR*: 16  *UTR*: 16  NS | Selection: ****  Comparability: -  Predictor: ***  7 MODERATE |
| 39 | Joober (2005)  “Increased prevalence of schizophrenia spectrum disorders in relatives of neuroleptic-nonresponsive schizophrenic patients” | Heritability  71 (35, 36)  Canada | An absence of remission of psychotic symptoms within the past 2 years. In the preceding 5 years, had undergone at least 3 periods of treatment with conventional neuroleptics from at least two distinct families of drugs at a dose equal to, or greater than, 750 mg chlorpromazine (CPZ) equivalents on monotherapy, or 1000 mg CPZ equivalents when a combination of neuroleptics was used for a continuous period of at least 6 weeks, and which resulted in no significant decrease in symptoms. Unable to function without supervision in all, or nearly all, domains of social and vocational activities and had a Global Assessment Score (GAS)_<40 within the last 12 months. | At least one hospitalization to a psychiatric institution because of an acute psychotic episode. During each hospitalization, patients experienced a full or partial remission in response to treatment with conventional neuroleptics within 6–8 weeks of continuous treatment. Able to function autonomously with only occasional supervision in all, or nearly all, domains of social and vocational activities. Never had to be admitted to hospital because of psychotic exacerbation while under continuous neuroleptic treatment. At least one psychotic relapse when neuroleptic medication was reduced or discontinued. Remission was defined as a complete or quasi complete disappearance of schizophrenic symptoms, with limited residual symptoms. At the time of enrolment, total BPRS scores were less than 30 with no more than one item scoring 4 and/or a CGI score less than 3 (borderline mentally ill). | Morbid risk of schizophrenia, long term psychiatric care and DSM-IV cluster A personality disorders in relatives (1^st^ degree relatives and 2^nd^ degree relatives) and familial loading score | **Morbid Risk**  **Schizophrenia**  (based on best-estimate family history diagnosis)  ***1^st^ degree relatives***  *AR*: 3.06 (5/163)  *TR*: 8.84 (13/147)  p=0.05  ***2^nd^ degree relatives***  *AR*: 0.75 (4/529)  *TR*: 2.81 (13/462)  p 0.02  **Long-term Psychiatric Care**  ***1^st^ degree relatives***  *AR:* 1.5 (3/200)  *TR:* 2.8 (5/178)  NS  ***2^nd^ degree relatives***  *AR*: 0.48 (3/627)  *TR*: 0.96 (5/522)  NS  **DSM-IV cluster A personality disorder**  ***1^st^ degree relatives***  *AR*: 3.51 (7/199)  *TR*: 5.71 (10/175)  NS  ***2^nd^ degree relatives***  *AR*: 0.84 (5/595)  *TR*: 1.01 (5/495)  NS  **Familial Loading**  Difference in scores  p=0.033  **Percentage of patients in FL score categories %**  **FL scores ≥ 4**  *AR*: 2.7  *TR*: 16.6  **FL scores <4 ≥ -0.5**  *AR*: 11.4  *TR*: 13.9  **FL scores < -0.5**  *AR*: 85.7  *TR*: 69.4 | Selection: ***  Comparability: -  Predictor: ****  7 MODERATE |
| 40 | Kayo (2012)  “Does lack of improvement in the first two weeks  predict treatment resistance in recent-onset  psychosis?” | Open, randomized prospective  17 (4, 13)  Brazil | IPAP algorithm, failed to respond (a >30% decrease in the PANSS  Score) to either of  two 4- to 6-week trials | Those that showed a >30% decrease in the PANSS  score at any time in the study | Baseline PANSS scores  Improvement at 2 weeks | **Lack of improvement at 2 weeks**  x2=0.60, df=1, p=0.57 NS  F=1.907, p=0.192 NS  **Baseline PANSS scores**  *AR*: 91.23  *TR*: 79.25  p=0.02 | Selection: ***  Comparability: -  Predictor: ****  7 MODERATE |
| 41 | Meltzer (1997)  “Age at onset and gender of schizophrenic patients in relation to neuroleptic resistance” | Case-control  322 (196, 126)  America | Kane criteria, including responders to clozapine. | Had at most mild positive and negative symptoms during most recent course of neuroleptic treatment. | Age of onset and ethnicity | **Age of onset**  *AR*: 22.5 (7.4) (F: 24.2 (8.7), M: 21.2 (6.1))  *TR*: 19.7 (5.3) (F: 20.1 (6.3), M: 19.4 (4.7))  F=17.1, df=1, 318, p<0.0001.  **Gender %**  *AR*: F: 40.48, M: 59.52  *TR*: F: 39.29, M: 60.71  NS  **Ethnicity**  **(AA Africa-American,**  **C Caucasian) %**  *AR*: AA: 30.95, C: 65.89  *TR*: AA: 13.27, C: 85.71  χ2=114.1, df=1, p=0.0002 | Selection: ****  Comparability: -  Predictor: ****  8 MODERATE |
| 42 | de Bartolomeis et al (2013)  “Differential cognitive performances between schizophrenic responders and non-responders to antipsychotics: correlation with course of the illness, psychopathology, attitude to the treatment and antipsychotics doses” | Cross sectional  41 (19,22)  Italy | He/she had failed to respond to two or three  trials with antipsychotic agents, given at therapeutic doses and for at least 6 weeks. | “those substantially compensated by an antipsychotic therapy”* | (1) Verbal memory by the List Learning task.  (2)Working memory by the Digit Sequencing task.  (3) Motor speed by the Token  Motor task.  (4) Verbal fluency by the Category Instances task. (5) Processing speed (i.e.  attention and speed of information processing) by the Symbol Coding task.  (6) Executive functions by the Tower of London task. | **Verbal Memory** (mean)  *AR*: ~2.2  *TR*: ~0.8  F=20.78, d.f.=2,60,  P<0.0001  **Working Memory**  *AR*: ~1.5  *TR*: ~1  NS  **Motor Speed**  *AR*: ~0.2  *TR*: 0  NS  **Verbal Fluency**  *AR*: ~1.3  *TR*: ~1  NS  **Processing Speed**  *AR*: ~0.7  *TR*: ~0.2  NS  **Executive Functions**  *AR*: ~ 1.3  *TR*: ~0.7  NS  (Numbers are not given, so means are based on figure provided, Figure 1, p389) | Selection: ****  Comparability: -  Predictor: ***  7 MODERATE |
| 43 | Joober (2002)  “Neuropsychological impairments in neuroleptic-responder vs. -nonresponder schizophrenic patients and healthy volunteers” | Cross-sectional  75 (39,36)  Canada | No remission of psychotic symptoms within the past two years. In preceding five years, all had undergone at least three periods of treatment with conventional neuroleptics from at least two distinct families of drugs at a dose equal to or greater than 750mg chlorpromazine equivalents on monotherapy or 1000mg equivalent, for a continuous period of at least six weeks and which resulted in no significant symptomatic improvement. Minimum score of 4 on at least five BPRS items, a total BPRS score of at least 45 and/or CGI score of at least 5. | Admitted at least once to psychiatric institution because of acute psychotic episode. Each hospitalisation, full or partial remission in response to treatment with conventional neuroleptics within 6-8 weeks of continuous. No admissions because of worsening while on continuous treatment. At least one relapse (worsening in symptoms requiring hospital or increased dose) when medication decreased or discontinued. Remission defined as complete or quasi complete disappearance of symptoms, limited residual symptoms, based on treating psychiatrist clinical evaluation and hospital record. Total BPRS less than 30, no more than 1 item scoring 4 and/or CGI score less than 3. | Neuropsychological domains (Sustained attention, abstraction and concept formation, visual spatial ability, visual-motor processing, visual memory, general verbal ability and language, and verbal memory and learning) | **Sustained attention**  F= 1.6, p = 0.2, NS  **Abstraction and concept formation**  F=4.7, p=0.03, NS with Bonferroni correction  **Visual spatial ability**  F=0.9, p=0.32, NS  **Visual-motor processing**  F=1.1, p=0.3, NS  **Visual memory**  F=9.2, p=0.003  **General verbal ability and language**  F=7.7, p = 0.007  **Verbal memory and learning**  F=54.9, p<.001 | Selection: ***  Comparability: -  Predictor: ****  7 MODERATE |

**Appendix VI - Quality Assessment for Included Studies**

| **Study 28: Anderson et al. 2015.** | | | | | | | | |
| --- | --- | --- | --- | --- | --- | --- | --- | --- |
| **SELECTION (max 5)** | | | | | **COMPARABILITY (max 2)** | **PREDICTOR (max 4)** | | |
| **1) Ascertainment of TR diagnosis** | **2) Ascertainment of AR diagnosis** | **3)**  **Selection of TR patients** | **4)**  **Selection of AR patients** | **5)**  **Sample size** | **Comparability on basis of design or analysis** | **1) Ascertainment of predictor** | **2)**  **Same method of ascertainment for TR and AR** | **3)**  **Statistical test** |
| a) Independent assessment.*  b) Independent review of records.*  c) Record linkage.  d) Self report.  e) No description. | a) Confirmed response to antipsychotic treatment, with experimental validation.*  b) Confirmed response to antipsychotic treatment, interview, records or self-report.*  c) No description. | a) All eligible cases with outcome of interest over a defined period of time, in defined population etc.*  b) An appropriate sample of those cases (e.g. random sample).*  c) Neither of the above, or not stated. | a) Derived from same population as TR, would have been TR if had treatment-resistance.*  b) Different source/different inclusion and exclusion.  c) No / inadequate description. | a) Justified and satisfactory.*  b) Not justified. | a) Study controls for age and sex.*  b) Study controls for any additional factor.* | a) Validated measurement tool, blind to TR/AR status.**  b) Validated, objective measurement tool, not blind to TR/AR status.*  c) Non-validated/ non-objective measurement tool, self report or medical record only.  d) No description. | a) Yes.*  b) No. | a) The statistical test used to analyze the data is clearly described and appropriate, and the measurement of the association is presented, and the probability level (p value).*  b) The statistical test is not appropriate, not described or incomplete. |

**Selection: *****

**Comparability: ***

**Predictor: *****

**7 MODERATE**

| **Study 29: Molina et al. 2008.** | | | | | | | | |
| --- | --- | --- | --- | --- | --- | --- | --- | --- |
| **SELECTION (max 5)** | | | | | **COMPARABILITY (max 2)** | **PREDICTOR (max 4)** | | |
| **1) Ascertainment of TR diagnosis** | **2) Ascertainment of AR diagnosis** | **3)**  **Selection of TR patients** | **4)**  **Selection of AR patients** | **5)**  **Sample size** | **Comparability on basis of design or analysis** | **1) Ascertainment of predictor** | **2)**  **Same method of ascertainment for TR and AR** | **3)**  **Statistical test** |
| a) Independent assessment.*  b) Independent review of records.*  c) Record linkage.  d) Self report.  e) No description. | a) Confirmed response to antipsychotic treatment, with experimental validation.*  b) Confirmed response to antipsychotic treatment, interview, records or self-report.*  c) No description. | a) All eligible cases with outcome of interest over a defined period of time, in defined population etc.*  b) An appropriate sample of those cases (e.g. random sample).*  c) Neither of the above, or not stated. | a) Derived from same population as TR, would have been TR if had treatment-resistance.*  b) Different source/different inclusion and exclusion.  c) No / inadequate description. | a) Justified and satisfactory.*  b) Not justified. | a) Study controls for **sex.***  b) Study controls for any additional factor.* | a) Validated measurement tool, blind to TR/AR status.**  b) Validated, objective measurement tool, not blind to TR/AR status.*  c) Non-validated/ non-objective measurement tool, self report or medical record only.  d) No description. | a) Yes.*  b) No. | a) The statistical test used to analyze the data is clearly described and appropriate, and the measurement of the association is presented, and the probability level (p value).*  b) The statistical test is not appropriate, not described or incomplete. |

**Selection: *****

**Comparability: ***

**Predictor: *****

**7 MODERATE**

| **Study 17: Demjaha et al. 2012.** | | | | | | | | |
| --- | --- | --- | --- | --- | --- | --- | --- | --- |
| **SELECTION (max 5)** | | | | | **COMPARABILITY (max 2)** | **PREDICTOR (max 4)** | | |
| **1) Ascertainment of TR diagnosis** | **2) Ascertainment of AR diagnosis** | **3)**  **Selection of TR patients** | **4)**  **Selection of AR patients** | **5)**  **Sample size** | **Comparability on basis of design or analysis** | **1) Ascertainment of predictor** | **2)**  **Same method of ascertainment for TR and AR** | **3)**  **Statistical test** |
| a) Independent assessment.*  b) Independent review of records.*  c) Record linkage.  d) Self report.  e) No description. | a) Confirmed response to antipsychotic treatment, with experimental validation.*  b) Confirmed response to antipsychotic treatment, interview, records or self-report.*  c) No description. | a) All eligible cases with outcome of interest over a defined period of time, in defined population etc.*  b) An appropriate sample of those cases (e.g. random sample).*  c) Neither of the above, or not stated. | a) Derived from same population as TR, would have been TR if had treatment-resistance.*  b) Different source/different inclusion and exclusion.  c) No / inadequate description. | a) Justified and satisfactory.*  b) Not justified. | a) Study controls for antipsychotic dosage and duration of illness.*  b) Study controls for any additional factor [age, gender, ethnicity, weight and smoking].* | a) Validated measurement tool, blind to TR/AR status.**  b) Validated, objective measurement tool, not blind to TR/AR status.*  c) Non-validated/ non-objective measurement tool, self report or medical record only.  d) No description. | a) Yes.*  b) No. | a) The statistical test used to analyze the data is clearly described and appropriate, and the measurement of the association is presented, and the probability level (p value).*  b) The statistical test is not appropriate, not described or incomplete. |

**Selection: *****

**Comparability: ****

**Predictor: *****

**8 MODERATE**

| **Study 18: Demjaha et al. 2014.** | | | | | | | | |
| --- | --- | --- | --- | --- | --- | --- | --- | --- |
| **SELECTION (max 5)** | | | | | **COMPARABILITY (max 2)** | **PREDICTOR (max 4)** | | |
| **1) Ascertainment of TR diagnosis** | **2) Ascertainment of AR diagnosis** | **3)**  **Selection of TR patients** | **4)**  **Selection of AR patients** | **5)**  **Sample size** | **Comparability on basis of design or analysis** | **1) Ascertainment of predictor** | **2)**  **Same method of ascertainment for TR and AR** | **3)**  **Statistical test** |
| a) Independent assessment.*  b) Independent review of records.*  c) Record linkage.  d) Self report.  e) No description. | a) Confirmed response to antipsychotic treatment, with experimental validation.*  b) Confirmed response to antipsychotic treatment, interview, records or self-report.*  c) No description. | a) All eligible cases with outcome of interest over a defined period of time, in defined population etc.*  b) An appropriate sample of those cases (e.g. random sample).*  c) Neither of the above, or not stated. | a) Derived from same population as TR, would have been TR if had treatment-resistance.*  b) Different source/different inclusion and exclusion.  c) No / inadequate description. | a) Justified and satisfactory.*  b) Not justified. | a) Study controls for antipsychotic dosage and duration of illness.*  b) Study controls for any additional factor [age, gender, ethnicity, weight and smoking].* | a) Validated measurement tool, blind to TR/AR status.**  b) Validated, objective measurement tool, not blind to TR/AR status.*  c) Non-validated/ non-objective measurement tool, self report or medical record only.  d) No description. | a) Yes.*  b) No. | a) The statistical test used to analyze the data is clearly described and appropriate, and the measurement of the association is presented, and the probability level (p value).*  b) The statistical test is not appropriate, not described or incomplete. |

**Selection: ****

**Comparability: ****

**Predictor: *****

**7 MODERATE**

| **Study 27: Goldstein et al. 2015.** | | | | | | | | |
| --- | --- | --- | --- | --- | --- | --- | --- | --- |
| **SELECTION (max 5)** | | | | | **COMPARABILITY (max 2)** | **PREDICTOR (max 4)** | | |
| **1) Ascertainment of TR diagnosis** | **2) Ascertainment of AR diagnosis** | **3)**  **Selection of TR patients** | **4)**  **Selection of AR patients** | **5)**  **Sample size** | **Comparability on basis of design or analysis** | **1) Ascertainment of predictor** | **2)**  **Same method of ascertainment for TR and AR** | **3)**  **Statistical test** |
| a) Independent assessment.*  b) Independent review of records.*  c) Record linkage.  d) Self report.  e) No description. | a) Confirmed response to antipsychotic treatment, with experimental validation.*  b) Confirmed response to antipsychotic treatment, interview, records or self-report.*  c) No description. | a) All eligible cases with outcome of interest over a defined period of time, in defined population etc.*  b) An appropriate sample of those cases (e.g. random sample).*  c) Neither of the above, or not stated. | a) Derived from same population as TR, would have been TR if had treatment-resistance.*  b) Different source/different inclusion and exclusion.  c) No / inadequate description. | a) Justified and satisfactory.*  b) Not justified. | a) Study controls for age.*  b) Study controls for any additional factor (years of education, smoking status and a positive test for THC).* | a) Validated measurement tool, blind to TR/AR status.**  b) Validated, objective measurement tool, not blind to TR/AR status.*  c) Non-validated/ non-objective measurement tool, self report or medical record only.  d) No description. | a) Yes.*  b) No. | a) The statistical test used to analyze the data is clearly described and appropriate, and the measurement of the association is presented, and the probability level (p value).*  b) The statistical test is not appropriate, not described or incomplete. |

**Selection: *****

**Comparability: ****

**Predictor: *****

**8 MODERATE**

| **Study 30: Anttila et al. 2005.** | | | | | | | | |
| --- | --- | --- | --- | --- | --- | --- | --- | --- |
| **SELECTION (max 5)** | | | | | **COMPARABILITY (max 2)** | **PREDICTOR (max 4)** | | |
| **1) Ascertainment of TR diagnosis** | **2) Ascertainment of AR diagnosis** | **3)**  **Selection of TR patients** | **4)**  **Selection of AR patients** | **5)**  **Sample size** | **Comparability on basis of design or analysis** | **1) Ascertainment of predictor** | **2)**  **Same method of ascertainment for TR and AR** | **3)**  **Statistical test** |
| a) Independent assessment.*  b) Independent review of records.*  c) Record linkage.  d) Self report.  e) No description. | a) Confirmed response to antipsychotic treatment, with experimental validation.*  b) Confirmed response to antipsychotic treatment, interview, records or self-report.*  c) No description. | a) All eligible cases with outcome of interest over a defined period of time, in defined population etc.*  b) An appropriate sample of those cases (e.g. random sample).*  c) Neither of the above, or not stated. | a) Derived from same population as TR, would have been TR if had treatment-resistance.*  b) Different source/different inclusion and exclusion.  c) No / inadequate description. | a) Justified and satisfactory.*  b) Not justified. | a) Study controls for _____________*  b) Study controls for any additional factor.* | a) Validated measurement tool, blind to TR/AR status.**  b) Validated, objective measurement tool, not blind to TR/AR status.*  c) Non-validated/ non-objective measurement tool, self report or medical record only.  d) No description. | a) Yes.*  b) No. | a) The statistical test used to analyze the data is clearly described and appropriate, and the measurement of the association is presented, and the probability level (p value).*  b) The statistical test is not appropriate, not described or incomplete. |

**Selection: ******

**Comparability: -**

**Predictor: *****

**7 MODERATE**

| **Study 33: Anttila et al. 2007.** | | | | | | | | |
| --- | --- | --- | --- | --- | --- | --- | --- | --- |
| **SELECTION (max 5)** | | | | | **COMPARABILITY (max 2)** | **PREDICTOR (max 4)** | | |
| **1) Ascertainment of TR diagnosis** | **2) Ascertainment of AR diagnosis** | **3)**  **Selection of TR patients** | **4)**  **Selection of AR patients** | **5)**  **Sample size** | **Comparability on basis of design or analysis** | **1) Ascertainment of predictor** | **2)**  **Same method of ascertainment for TR and AR** | **3)**  **Statistical test** |
| a) Independent assessment.*  b) Independent review of records.*  c) Record linkage.  d) Self report.  e) No description. | a) Confirmed response to antipsychotic treatment, with experimental validation.*  b) Confirmed response to antipsychotic treatment, interview, records or self-report.*  c) No description. | a) All eligible cases with outcome of interest over a defined period of time, in defined population etc.*  b) An appropriate sample of those cases (e.g. random sample).*  c) Neither of the above, or not stated. | a) Derived from same population as TR, would have been TR if had treatment-resistance.*  b) Different source/different inclusion and exclusion.  c) No / inadequate description. | a) Justified and satisfactory. *  b) Not justified. | a) Study controls for _______________*  b) Study controls for any additional factor.* | a) Validated measurement tool, blind to TR/AR status.**  b) Validated, objective measurement tool, not blind to TR/AR status.*  c) Non-validated/ non-objective measurement tool, self report or medical record only.  d) No description. | a) Yes.*  b) No. | a) The statistical test used to analyze the data is clearly described and appropriate, and the measurement of the association is presented, and the probability level (p value).*  b) The statistical test is not appropriate, not described or incomplete. |

**Selection: *****

**Comparability: -**

**Predictor: ******

**7 MODERATE**

| **Study 34: Goldberger et al. 2005.** | | | | | | | | |
| --- | --- | --- | --- | --- | --- | --- | --- | --- |
| **SELECTION (max 5)** | | | | | **COMPARABILITY (max 2)** | **PREDICTOR (max 4)** | | |
| **1) Ascertainment of TR diagnosis** | **2) Ascertainment of AR diagnosis** | **3)**  **Selection of TR patients** | **4)**  **Selection of AR patients** | **5)**  **Sample size** | **Comparability on basis of design or analysis** | **1) Ascertainment of predictor** | **2)**  **Same method of ascertainment for TR and AR** | **3)**  **Statistical test** |
| a) Independent assessment.*  b) Independent review of records.*  c) Record linkage.  d) Self report.  e) No description. | a) Confirmed response to antipsychotic treatment, with experimental validation.*  b) Confirmed response to antipsychotic treatment, interview, records or self-report.*  c) No description. | a) All eligible cases with outcome of interest over a defined period of time, in defined population etc.*  b) An appropriate sample of those cases (e.g. random sample).*  c) Neither of the above, or not stated. | a) Derived from same population as TR, would have been TR if had treatment-resistance.*  b) Different source/different inclusion and exclusion.  c) No / inadequate description. | a) Justified and satisfactory.*  b) Not justified. | a) Study controls for _______________.*  b) Study controls for any additional factor.* | a) Validated measurement tool, blind to TR/AR status.**  b) Validated, objective measurement tool, not blind to TR/AR status.*  c) Non-validated/ non-objective measurement tool, self report or medical record only.  d) No description. | a) Yes.*  b) No. | a) The statistical test used to analyze the data is clearly described and appropriate, and the measurement of the association is presented, and the probability level (p value).*  b) The statistical test is not appropriate, not described or incomplete. |

**Selection: *****

**Comparability: -**

**Predictor: *****

**6 MODERATE**

| **Study 32: Joober et al. 1999.** | | | | | | | | |
| --- | --- | --- | --- | --- | --- | --- | --- | --- |
| **SELECTION (max 5)** | | | | | **COMPARABILITY (max 2)** | **PREDICTOR (max 4)** | | |
| **1) Ascertainment of TR diagnosis** | **2) Ascertainment of AR diagnosis** | **3)**  **Selection of TR patients** | **4)**  **Selection of AR patients** | **5)**  **Sample size** | **Comparability on basis of design or analysis** | **1) Ascertainment of predictor** | **2)**  **Same method of ascertainment for TR and AR** | **3)**  **Statistical test** |
| a) Independent assessment.*  b) Independent review of records.*  c) Record linkage.  d) Self report.  e) No description. | a) Confirmed response to antipsychotic treatment, with experimental validation.*  b) Confirmed response to antipsychotic treatment, interview, records or self-report.*  c) No description. | a) All eligible cases with outcome of interest over a defined period of time, in defined population etc.*  b) An appropriate sample of those cases (e.g. random sample).*  c) Neither of the above, or not stated. | a) Derived from same population as TR, would have been TR if had treatment-resistance.*  b) Different source/different inclusion and exclusion.  c) No / inadequate description. | a) Justified and satisfactory.*  b) Not justified. | a) Study controls for _______________*  b) Study controls for any additional factor.* | a) Validated measurement tool, blind to TR/AR status.**  b) Validated, objective measurement tool, not blind to TR/AR status.*  c) Non-validated/ non-objective measurement tool, self report or medical record only.  d) No description. | a) Yes.*  b) No. | a) The statistical test used to analyze the data is clearly described and appropriate, and the measurement of the association is presented, and the probability level (p value).*  b) The statistical test is not appropriate, not described or incomplete. |

**Selection: *****

**Comparability: -**

**Predictor: ******

**7 MODERATE**

| **Study 35: Kampman et al. 2006.** | | | | | | | | |
| --- | --- | --- | --- | --- | --- | --- | --- | --- |
| **SELECTION (max 5)** | | | | | **COMPARABILITY (max 2)** | **PREDICTOR (max 4)** | | |
| **1) Ascertainment of TR diagnosis** | **2) Ascertainment of AR diagnosis** | **3)**  **Selection of TR patients** | **4)**  **Selection of AR patients** | **5)**  **Sample size** | **Comparability on basis of design or analysis** | **1) Ascertainment of predictor** | **2)**  **Same method of ascertainment for TR and AR** | **3)**  **Statistical test** |
| a) Independent assessment.*  b) Independent review of records.*  c) Record linkage.  d) Self report.  e) No description. | a) Confirmed response to antipsychotic treatment, with experimental validation.*  b) Confirmed response to antipsychotic treatment, interview, records or self-report.*  c) No description. | a) All eligible cases with outcome of interest over a defined period of time, in defined population etc.*  b) An appropriate sample of those cases (e.g. random sample).*  c) Neither of the above, or not stated. | a) Derived from same population as TR, would have been TR if had treatment-resistance.*  b) Different source/different inclusion and exclusion.  c) No / inadequate description. | a) Justified and satisfactory. *  b) Not justified. | a) Study controls for _______________ *  b) Study controls for any additional factor.* | a) Validated measurement tool, blind to TR/AR status.**  b) Validated, objective measurement tool, not blind to TR/AR status.*  c) Non-validated/ non-objective measurement tool, self report or medical record only.  d) No description. | a) Yes.*  b) No. | a) The statistical test used to analyze the data is clearly described and appropriate, and the measurement of the association is presented, and the probability level (p value).*  b) The statistical test is not appropriate, not described or incomplete. |

**Selection: *****

**Comparability: -**

**Predictor *****

**6 MODERATE**

| **Study 36: Krebs et al. 1998.** | | | | | | | | |
| --- | --- | --- | --- | --- | --- | --- | --- | --- |
| **SELECTION (max 5)** | | | | | **COMPARABILITY (max 2)** | **PREDICTOR (max 4)** | | |
| **1) Ascertainment of TR diagnosis** | **2) Ascertainment of AR diagnosis** | **3)**  **Selection of TR patients** | **4)**  **Selection of AR patients** | **5)**  **Sample size** | **Comparability on basis of design or analysis** | **1) Ascertainment of predictor** | **2)**  **Same method of ascertainment for TR and AR** | **3)**  **Statistical test** |
| a) Independent assessment.*  b) Independent review of records.*  c) Record linkage.  d) Self report.  e) No description. | a) Confirmed response to antipsychotic treatment, with experimental validation.*  b) Confirmed response to antipsychotic treatment, interview, records or self-report.*  c) No description. | a) All eligible cases with outcome of interest over a defined period of time, in defined population etc.*  b) An appropriate sample of those cases (e.g. random sample).*  c) Neither of the above, or not stated. | a) Derived from same population as TR, would have been TR if had treatment-resistance.*  b) Different source/different inclusion and exclusion.  c) No/inadequate description. | a) Justified and satisfactory.*  b) Not justified. | a) Study controls for _______________ *  b) Study controls for any additional factor.* | a) Validated measurement tool, blind to TR/AR status.**  b) Validated, objective measurement tool, not blind to TR/AR status.*  c) Non-validated/ non-objective measurement tool, self report or medical record only.  d) No description. | a) Yes.*  b) No. | a) The statistical test used to analyze the data is clearly described and appropriate, and the measurement of the association is presented, and the probability level (p value).*  b) The statistical test is not appropriate, not described or incomplete. |

**Selection: ****

**Comparability: -**

**Predictor: ******

**6 MODERATE**

| **Study 31: Krebs et al. 2000.** | | | | | | | | |
| --- | --- | --- | --- | --- | --- | --- | --- | --- |
| **SELECTION (max 5)** | | | | | **COMPARABILITY (max 2)** | **PREDICTOR (max 4)** | | |
| **1) Ascertainment of TR diagnosis** | **2) Ascertainment of AR diagnosis** | **3)**  **Selection of TR patients** | **4)**  **Selection of AR patients** | **5)**  **Sample size** | **Comparability on basis of design or analysis** | **1) Ascertainment of predictor** | **2)**  **Same method of ascertainment for TR and AR** | **3)**  **Statistical test** |
| a) Independent assessment.*  b) Independent review of records.*  c) Record linkage.  d) Self report.  e) No description. | a) Confirmed response to antipsychotic treatment, with experimental validation.*  b) Confirmed response to antipsychotic treatment, interview, records or self-report.*  c) No description. | a) All eligible cases with outcome of interest over a defined period of time, in defined population etc.*  b) An appropriate sample of those cases (e.g. random sample).*  c) Neither of the above, or not stated. | a) Derived from same population as TR, would have been TR if had treatment-resistance.*  b) Different source/different inclusion and exclusion.  c) No / inadequate description. | a) Justified and satisfactory.*  b) Not justified. | a) Study controls for _______________ *  b) Study controls for any additional factor* | a) Validated measurement tool, blind to TR/AR status.**  b) Validated, objective measurement tool, not blind to TR/AR status.*  c) Non-validated/ non-objective measurement tool, self report or medical record only.  d) No description. | a) Yes.*  b) No. | a) The statistical test used to analyze the data is clearly described and appropriate, and the measurement of the association is presented, and the probability level (p value).*  b) The statistical test is not appropriate, not described or incomplete. |

**Selection: ****

**Comparability: -**

**Predictor: ******

**6 MODERATE**

| **Study 37: Lahdelma et al. 1998.** | | | | | | | | |
| --- | --- | --- | --- | --- | --- | --- | --- | --- |
| **SELECTION (max 5)** | | | | | **COMPARABILITY (max 2)** | **PREDICTOR (max 4)** | | |
| **1) Ascertainment of TR diagnosis** | **2) Ascertainment of AR diagnosis** | **3)**  **Selection of TR patients** | **4)**  **Selection of AR patients** | **5)**  **Sample size** | **Comparability on basis of design or analysis** | **1) Ascertainment of predictor** | **2)**  **Same method of ascertainment for TR and AR** | **3)**  **Statistical test** |
| a) Independent assessment.*  b) Independent review of records.*  c) Record linkage.  d) Self report.  e) No description. | a) Confirmed response to antipsychotic treatment, with experimental validation.*  b) Confirmed response to antipsychotic treatment, interview, records or self-report.*  c) No description. | a) All eligible cases with outcome of interest over a defined period of time, in defined population etc.*  b) An appropriate sample of those cases (e.g. random sample).*  c) Neither of the above, or not stated. | a) Derived from same population as TR, would have been TR if had treatment-resistance.*  b) Different source/different inclusion and exclusion.  c) No / inadequate description. | a) Justified and satisfactory.*  b) Not justified. | a) Study controls for _______________ *  b) Study controls for any additional factor.* | a) Validated measurement tool, blind to TR/AR status.**  b) Validated, objective measurement tool, not blind to TR/AR status.*  c) Non-validated/ non-objective measurement tool, self report or medical record only.  d) No description. | a) Yes.*  b) No. | a) The statistical test used to analyze the data is clearly described and appropriate, and the measurement of the association is presented, and the probability level (p value).*  b) The statistical test is not appropriate, not described or incomplete. |

**Selection: ******

**Comparability: -**

**Predictor: *****

**7 MODERATE**

| **Study 38: Meged et al. 1999.** | | | | | | | | |
| --- | --- | --- | --- | --- | --- | --- | --- | --- |
| **SELECTION (max 5)** | | | | | **COMPARABILITY (max 2)** | **PREDICTOR (max 4)** | | |
| **1) Ascertainment of TR diagnosis** | **2) Ascertainment of AR diagnosis** | **3)**  **Selection of TR patients** | **4)**  **Selection of AR patients** | **5)**  **Sample size** | **Comparability on basis of design or analysis** | **1) Ascertainment of predictor** | **2)**  **Same method of ascertainment for TR and AR** | **3)**  **Statistical test** |
| a) Independent assessment.*  b) Independent review of records.*  c) Record linkage.  d) Self report.  e) No description. | a) Confirmed response to antipsychotic treatment, with experimental validation.*  b) Confirmed response to antipsychotic treatment, interview, records or self-report.*  c) No description. | a) All eligible cases with outcome of interest over a defined period of time, in defined population etc.*  b) An appropriate sample of those cases (e.g. random sample).*  c) Neither of the above, or not stated. | a) Derived from same population as TR, would have been TR if had treatment-resistance.*  b) Different source/different inclusion and exclusion.  c) No / inadequate description. | a) Justified and satisfactory.*  b) Not justified. | a) Study controls for _______________ *  b) Study controls for any additional factor.* | a) Validated measurement tool, blind to TR/AR status.**  b) Validated, objective measurement tool, not blind to TR/AR status.*  c) Non-validated/ non-objective measurement tool, self report or medical record only.  d) No description. | a) Yes.*  b) No. | a) The statistical test used to analyze the data is clearly described and appropriate, and the measurement of the association is presented, and the probability level (p value).*  b) The statistical test is not appropriate, not described or incomplete. |

**Selection: ******

**Comparability: -**

**Predictor: *****

**7 MODERATE**

| **Study 39: Joober et al. 2005.** | | | | | | | | |
| --- | --- | --- | --- | --- | --- | --- | --- | --- |
| **SELECTION (max 5)** | | | | | **COMPARABILITY (max 2)** | **PREDICTOR (max 4)** | | |
| **1) Ascertainment of TR diagnosis** | **2) Ascertainment of AR diagnosis** | **3)**  **Selection of TR patients** | **4)**  **Selection of AR patients** | **5)**  **Sample size** | **Comparability on basis of design or analysis** | **1) Ascertainment of predictor** | **2)**  **Same method of ascertainment for TR and AR** | **3)**  **Statistical test** |
| a) Independent assessment.*  b) Independent review of records.*  c) Record linkage.  d) Self report.  e) No description. | a) Confirmed response to antipsychotic treatment, with experimental validation.*  b) Confirmed response to antipsychotic treatment, interview, records or self-report.*  c) No description. | a) All eligible cases with outcome of interest over a defined period of time, in defined population etc.*  b) An appropriate sample of those cases (e.g. random sample).*  c) Neither of the above, or not stated. | a) Derived from same population as TR, would have been TR if had treatment-resistance.*  b) Different source/different inclusion and exclusion.  c) No / inadequate description. | a) Justified and satisfactory.*  b) Not justified. | a) Study controls for _______________ *  b) Study controls for any additional factor.* | a) Validated measurement tool, blind to TR/AR status.**  b) Validated, objective measurement tool, not blind to TR/AR status.*  c) Non-validated/ non-objective measurement tool, self report or medical record only.  d) No description. | a) Yes.*  b) No. | a) The statistical test used to analyze the data is clearly described and appropriate, and the measurement of the association is presented, and the probability level (p value).*  b) The statistical test is not appropriate, not described or incomplete. |

**Selection: *****

**Comparability: -**

**Predictor: ******

**7 MODERATE**

| **Study 40: Kayo et al. 2012.** | | | | | | | | |
| --- | --- | --- | --- | --- | --- | --- | --- | --- |
| **SELECTION (max 5)** | | | | | **COMPARABILITY (max 2)** | **PREDICTOR (max 4)** | | |
| **1) Ascertainment of TR diagnosis** | **2) Ascertainment of AR diagnosis** | **3)**  **Selection of TR patients** | **4)**  **Selection of AR patients** | **5)**  **Sample size** | **Comparability on basis of design or analysis** | **1) Ascertainment of predictor** | **2)**  **Same method of ascertainment for TR and AR** | **3)**  **Statistical test** |
| a) Independent assessment.*  b) Independent review of records.*  c) Record linkage.  d) Self report.  e) No description. | a) Confirmed response to antipsychotic treatment, with experimental validation.*  b) Confirmed response to antipsychotic treatment, interview, records or self-report.*  c) No description. | a) All eligible cases with outcome of interest over a defined period of time, in defined population etc.*  b) An appropriate sample of those cases (e.g. random sample).*  c) Neither of the above, or not stated. | a) Derived from same population as TR, would have been TR if had treatment-resistance.*  b) Different source/different inclusion and exclusion.  c) No / inadequate description. | a) Justified and satisfactory.*  b) Not justified. | a) Study controls for _______________ *  b) Study controls for any additional factor.* | a) Validated measurement tool, blind to TR/AR status.**  b) Validated, objective measurement tool, not blind to TR/AR status.*  c) Non-validated/ non-objective measurement tool, self report or medical record only.  d) No description. | a) Yes.*  b) No. | a) The statistical test used to analyze the data is clearly described and appropriate, and the measurement of the association is presented, and the probability level (p value).*  b) The statistical test is not appropriate, not described or incomplete. |

**Selection: *****

**Comparability: -**

**Predictor: ******

**7 MODERATE**

| **Study 41: Meltzer et al. 1997.** | | | | | | | | |
| --- | --- | --- | --- | --- | --- | --- | --- | --- |
| **SELECTION (max 5)** | | | | | **COMPARABILITY (max 2)** | **PREDICTOR (max 4)** | | |
| **1) Ascertainment of TR diagnosis** | **2) Ascertainment of AR diagnosis** | **3)**  **Selection of TR patients** | **4)**  **Selection of AR patients** | **5)**  **Sample size** | **Comparability on basis of design or analysis** | **1) Ascertainment of predictor** | **2)**  **Same method of ascertainment for TR and AR** | **3)**  **Statistical test** |
| a) Independent assessment.*  b) Independent review of records.*  c) Record linkage.  d) Self report.  e) No description. | a) Confirmed response to antipsychotic treatment, with experimental validation.*  b) Confirmed response to antipsychotic treatment, interview, records or self-report.*  c) No description. | a) All eligible cases with outcome of interest over a defined period of time, in defined population etc.*  b) An appropriate sample of those cases (e.g. random sample).*  c) Neither of the above, or not stated. | a) Derived from same population as TR, would have been TR if had treatment-resistance.*  b) Different source/different inclusion and exclusion.  c) No / inadequate description. | a) Justified and satisfactory.*  b) Not justified. | a) Study controls for _______________ *  b) Study controls for any additional factor.* | a) Validated measurement tool, blind to TR/AR status.**  b) Validated, objective measurement tool, not blind to TR/AR status.*  c) Non-validated/ non-objective measurement tool, self report or medical record only.  d) No description. | a) Yes.*  b) No. | a) The statistical test used to analyze the data is clearly described and appropriate, and the measurement of the association is presented, and the probability level (p value).*  b) The statistical test is not appropriate, not described or incomplete. |

**Selection: ******

**Comparability: -**

**Predictor: ******

**8 MODERATE**

| **Study 42: de Bartolomeis et al. 2013.** | | | | | | | | |
| --- | --- | --- | --- | --- | --- | --- | --- | --- |
| **SELECTION (max 5)** | | | | | **COMPARABILITY (max 2)** | **PREDICTOR (max 4)** | | |
| **1) Ascertainment of TR diagnosis** | **2) Ascertainment of AR diagnosis** | **3)**  **Selection of TR patients** | **4)**  **Selection of AR patients** | **5)**  **Sample size** | **Comparability on basis of design or analysis** | **1) Ascertainment of predictor** | **2)**  **Same method of ascertainment for TR and AR** | **3)**  **Statistical test** |
| a) Independent assessment.*  b) Independent review of records.*  c) Record linkage.  d) Self report.  e) No description. | a) Confirmed response to antipsychotic treatment, with experimental validation.*  b) Confirmed response to antipsychotic treatment, interview, records or self-report.*  c) No description. | a) All eligible cases with outcome of interest over a defined period of time, in defined population etc.*  b) An appropriate sample of those cases (e.g. random sample).*  c) Neither of the above, or not stated. | a) Derived from same population as TR, would have been TR if had treatment-resistance.*  b) Different source/different inclusion and exclusion.  c) No / inadequate description. | a) Justified and satisfactory.*  b) Not justified. | a) Study controls for _______________ *  b) Study controls for any additional factor.* | a) Validated measurement tool, blind to TR/AR status.**  b) Validated, objective measurement tool, not blind to TR/AR status.*  c) Non-validated/ non-objective measurement tool, self report or medical record only.  d) No description. | a) Yes.*  b) No. | a) The statistical test used to analyze the data is clearly described and appropriate, and the measurement of the association is presented, and the probability level (p value).*  b) The statistical test is not appropriate, not described or incomplete. |

**Selection: ******

**Comparability: -**

**Predictor: *****

**7 MODERATE**

| **Study 43: Joober et al. 2002.** | | | | | | | | |
| --- | --- | --- | --- | --- | --- | --- | --- | --- |
| **SELECTION (max 5)** | | | | | **COMPARABILITY (max 2)** | **PREDICTOR (max 4)** | | |
| **1) Ascertainment of TR diagnosis** | **2) Ascertainment of AR diagnosis** | **3)**  **Selection of TR patients** | **4)**  **Selection of AR patients** | **5)**  **Sample size** | **Comparability on basis of design or analysis** | **1) Ascertainment of predictor** | **2)**  **Same method of ascertainment for TR and AR** | **3)**  **Statistical test** |
| a) Independent assessment.*  b) Independent review of records.*  c) Record linkage.  d) Self report.  e) No description. | a) Confirmed response to antipsychotic treatment, with experimental validation.*  b) Confirmed response to antipsychotic treatment, interview, records or self-report.*  c) No description. | a) All eligible cases with outcome of interest over a defined period of time, in defined population etc.*  b) An appropriate sample of those cases (e.g. random sample).*  c) Neither of the above, or not stated. | a) Derived from same population as TR, would have been TR if had treatment-resistance.*  b) Different source/different inclusion and exclusion.  c) No / inadequate description. | a) Justified and satisfactory.*  b) Not justified. | a) Study controls for _______________ *  b) Study controls for any additional factor.* | a) Validated measurement tool, blind to TR/AR status.**  b) Validated, objective measurement tool, not blind to TR/AR status.*  c) Non-validated/ non-objective measurement tool, self report or medical record only.  d) No description. | a) Yes.*  b) No. | a) The statistical test used to analyze the data is clearly described and appropriate, and the measurement of the association is presented, and the probability level (p value).*  b) The statistical test is not appropriate, not described or incomplete. |

**Selection: *****

**Comparability: -**

**Predictor: ******

**7 MODERATE**
